# Use of Metabolomics Approach in the Discovery of Active Compounds from Macroalgae *Laurencia* Species Against Schistosomiasis

**DOI:** 10.3390/pharmaceutics17101294

**Published:** 2025-10-02

**Authors:** Amanda Beatriz Silva Soares, Patricia Aoki Miyasato, Rafaela Paula de Freitas, Adolfo Luis Almeida Maleski, Daniel Carvalho Pimenta, Pio Colepicolo, Erika Mattos Stein, Arthur Ladeira Macedo, Carlos Alexandre Carollo, Eliana Nakano

**Affiliations:** 1 Laboratory of Parasitology, Butantan Institute, São Paulo 05503-900, SP, Brazil; amanda.ssoares@icb.usp.br (A.B.S.S.);; 2 Laboratory of Biochemistry and Molecular Biology of Marine Algae, Chemistry Institute, University of Sao Paulo, São Paulo 05508-090, SP, Brazilstein.erika.m@gmail.com (E.M.S.); 3 Interunits Postgraduate Program in Biotechnology (PPIB), Institute of Biomedical Sciences, University of Sao Paulo, São Paulo 05508-000, SP, Brazil; 4 Experimental Morphophysiology Laboratory, Natural and Humanities Sciences Center (CCNH), Federal University of ABC (UFABC), São Bernardo do Campo 09606-070, SP, Brazil; 5 Laboratory of Biochemistry, Butantan Institute, São Paulo 05503-900, SP, Brazil; dcpimenta@butantan.gov.br; 6 Ecology and Evolution Laboratory, Butantan Institute, São Paulo 05503-900, SP, Brazil; 7 Laboratory of Natural Products and Mass Spectrometry—LaPNEM, Faculty of Pharmaceutical Sciences, Food and Nutrition, Federal University of Mato Grosso do Sul, Campo Grande 79070-900, MS, Brazilcarlos.carollo@ufms.br (C.A.C.)

**Keywords:** *Schistosoma mansoni*, schistosomiasis, metabolomic approach, marine natural products, red macroalgae, *Laurencia* complex

## Abstract

**Background**: Marine macroalgae has been studied by our research group as alternative sources of bioactive compounds with promising antiparasitic activity, particularly against *Schistosoma mansoni*. **Objectives:** This study aimed to employ a metabolomics-based approach to identify anthelminthic active compounds from the macroalgae *Laurencia aldingensis* Saito and Womersley 1974 and *Laurencia dendroidea* J. Agardh 1852. **Methods:** The algae were extracted using a dichloromethane/methanol mixture, followed by liquid–liquid partitioning and sequential chromatographic fractionation using solvents of varying polarities. The resulting fractions were tested for biological activity against adult *Schistosoma mansoni* worms. Detailed chemical characterization of the extracts was conducted via HPLC-DAD-MS/MS, with subsequent data alignment and statistical analysis (Pearson correlation) to associate specific chemical compounds with the observed bioactivity. **Results:** Non-polar fractions (hexane and dichloromethane) exhibited significant anthelminthic activity, substantially reducing parasite viability and reproduction. Specific subfractions obtained from the dichloromethane fraction demonstrated notable activity. Metabolomic analysis revealed considerable chemical diversity, emphasizing the presence of bromophenols and halogenated sesquiterpenes, including potentially novel compounds with therapeutic potential against schistosomiasis. **Conclusions:** The metabolomics approach proved effective in identifying promising bioactive compounds from *Laurencia* spp. macroalgae with activity against *S. mansoni*.

## 1. Introduction

Bioguided fractioning has been traditionally used in bioprospection studies to isolate and identify active compounds. Despite the simplicity and low cost, the technique is not always effective, especially if not directed to specific classes of already known active compounds. Crude extracts of natural origin are complex biological matrices with, not rarely, several active compounds, requiring sophisticated and advanced analytical methods to target bioactive metabolites [1]. The complexity of highlighting active metabolites may represent an even greater challenge for non-fully explored natural sources, such as marine macroalgae, when compared to plant species.

The potential of macroalgae as a source of bioactive compounds for neglected diseases has yet to be thoroughly explored [2]. Schistosomiasis remains an important public health problem affecting about 250 million people worldwide [3]. As for the other neglected diseases, the chemical arsenal to the control of schistosomiasis is limited to few drugs raising concern with the emergence of resistance [4,5]. Within this context, our group has been working on bioprospection studies with Brazilian macroalgae to identify compounds of potential use in the treatment and control of schistosomiasis. In a preliminary screening, 13 crude extracts were tested for activity on *Schistosoma mansoni* [6]. In a following and more comprehensive study, 45 crude extracts obtained from 37 species were screened for molluscicidal activity against *Biomphalaria glabrata* embryos and antischistosomal activity against *S. mansoni*, identifying 22 species (60%) with activity in at least one of the two models [7]. Species selected in the trial step were further assessed for the isolation and identification of active compounds through bioguided fractionation. Three halogenated monoterpenes and the sesquiterpene elatol were identified as the compounds responsible for the antiparasitic and molluscicidal activities observed for the red macroalgae *Ochtodes secundiramea* and *Laurencia dendroidea* [8,9]. These results highlight the potential of seaweeds as a source of novel bioactives for schistosomiasis control.

Alternatively to the bioguided fractionation, we employed a metabolomic approach to annotate active compounds directly from the crude extracts tested in the screening study on *S. mansoni* and *B. glabrata* [7]. The data from the GC–MS analysis along with those from the biological activity were subjected to correlation tests to point the main hits. However, due to the chemical complexity, the metabolomic analysis was performed with taxonomically related groups presenting at least three active extracts and three inactive/relatively inactive extracts, resulting in the establishment of two sets—Ochrophyta and *Laurencia*/*Laurenciella*. Therefore, the constitution of sets for statistical analysis allowed for the alignment of the data despite the chemical complexity of the species studied. In this way, we were able to analyze the chemical compositions and biological activities interdependence and identify the most promising hits. The Pattern Hunter test was used to list these hits, and the heatmap permitted a closer look at the distribution pattern of these compounds in the extracts, contributing to an initial classification of the promising hits.

For the present study, *Laurencia aldingensis* and *L. dendroidea*, Rhodophyta species which demonstrated to be a potential source of both antischistosomal and molluscicidal compounds and integrated the *Laurencia*/*Laurenciella* set in our previous studies were selected [7,9].

Aiming a more efficient and direct separation of metabolites from the complex macroalgal matrix in the pre-analytical stage, a meticulously refined extraction protocol was used. Moreover, the metabolomics-based approach allowed not only to point hits, but also to identify possible synergistic interactions among different classes of compounds.

## 2. Materials and Methods

### 2.1. Experimental Design

A crude extract was initially obtained with a dichloromethane/methanol (1:1, *v*/*v*) combination and submitted to a liquid–liquid partition. The resulting dichloromethane phase was submitted to a sequential fractionation based on the solvent’s polarities: hexane, dichloromethane, ethyl acetate, and methanol.

The resulting fractions were assessed for antischistosomal activity at 100, 75, 50, 25 µg/mL with praziquantel 1.5 μg/mL as positive control and DMSO 1.5% μg/mL as negative control. The antiparasitic activity was monitored for 96 h for the effects on motility and reproduction and classified based on a score system as: No effect (0–10), Poor (11–37), Regular (38–80), and Good (81–150).

The dichloromethane fractions of both species were subfractioned on a HPLC—LC RID and assessed for biological activity and analyzed by HPLC-MS/MS for metabolomic approach.

### 2.2. Algae Sampling

The algae samples of *Laurencia dendroidea* and *L. aldingensis* samples were in Espírito Santo State, Southeastern Brazil and a voucher of representative specimens were deposited at the Maria Eneyda P. Kauffmann Fidalgo Herbarium (SP) at the Instituto de Botânica in São Paulo—*L. dendroidea* (voucher: SP 399.936) and *L. aldingensis* (voucher: SP 400.203). After collection, the fresh algae were washed thoroughly with seawater to remove sand particles and epiphytes. The cleaned material was stored frozen in zip-lock plastic bags at −20 °C until preparation of the extracts.

### 2.3. Obtention of Extracts, Fractions and Subfractions

After lyophilization and grinding, extracts were obtained by maceration of the material with dichloromethane and methanol 1:1 for 48 h, filtered, and concentrated under reduced pressure. The process was repeated 5 times to the obtention of 1200 mL of crude extract from *L. dendroidea* and 600 mL from *L. aldingensis*. Next, a liquid–liquid partition was performed by adding ultrapure water at 1:1:1 to the obtention of the hydromethanolic and dichloromethane phase. After adsorbed in silica, the dichloromethane phase was submitted to a sequential fractioning with hexane, dichloromethane, ethyl acetate and methanol. A volume of 450 mL of each solvent was used for *L. dendroidea* and 200 mL for *L. aldingensis*; all fractions were concentrated on a rotary evaporator.

The dichloromethane fractions of both species were subfractioned on a HPLC—LC RID—20 (Shimadzu^®^, Kyoto, Japan) high efficiency liquid chromatograph with a reverse column C8 (250 × 4.6 mm × 5 µm). The mobile phase consisted of ultrapure water (solvent A) and acetonitrile 90% (solvent B) each mixed with 0.1% formic acid (*v*/*v*). trifluoracetic acid 0.1%. The gradient elution profile was programmed as follows: an initial period of 0–5 min with 40% solvent B, followed by a linear increase from 40% to 100% solvent B over 5–40 min, remaining at 100% solvent B between 40 and 50 min. Following a linear decay from 100% to 40% solvent B over 50–55 min. Post-gradient, the column underwent a 5 min wash and reconditioning phase. The flow rate was 1 mL/min, with an injection volume of 200 µL.

Detection parameters included ultraviolet (UV) monitoring at the wavelengths of 214–254 nm with manual injection and collection.

For *L. dendroidea*, 7 subfractions were obtained from 84.2 mg and 7 subfractions from 71.5 mg for *L. aldingensis*; fractions were collected 10 times.

### 2.4. HPLC-DAD-MS/MS Analyses

All extracts and fractions were initially solubilized in a methanol and water mixture (7:3, *v*/*v*) at uniform concentrations. Following solubilization, the solutions were filtered using a 0.22 µm PTFE syringe filter. Analytical procedures were conducted using a Shi-madzu LC-20AD system (Shimadzu, Kyoto, Japan), integrated with a diode array detector and an ESI-qTOF mass spectrometer (MicroTOF-Q III, Bruker Daltonics, Billerica, Billerica, MA, USA).

Chromatographic separation was achieved on a highly efficient Kinetex^®^ C-18 column (2.6 μm, 150 × 2.2 mm, Phenomenex^®^, Torrance, CA, USA), safeguarded by a corresponding pre-column. The column was maintained at 50 °C during the analysis. The mobile phase consisted of ultrapure water (solvent A) and acetonitrile (solvent B), each mixed with 0.1% formic acid (*v*/*v*).

The gradient elution profile was programmed as follows: an initial 0–2 min period with 3% solvent B, followed by a linear increase from 3% to 25% solvent B over 2–25 min, culminating in a ramp from 25% to 80% solvent B between 25 and 40 min. Post-gradient, the column underwent an 8 min washing and reconditioning phase. The flow rate was 0.3 mL/min, with an injection volume of 1 µL.

Detection parameters included ultraviolet (UV) monitoring across a broad wave-length range of 240 to 800 nm. The mass spectrometer operated in both negative and positive ionization modes, covering a mass-to-charge ratio (*m*/*z*) range of 120–1.200. A capillary voltage of 4500 V was applied, with nitrogen serving as the nebulizer gas at 4 bar and as the drying gas at a flow rate of 9 L/min. Data were processed using DataAnalysis 4.2 software (Bruker Daltonics, Billerica, MA, USA).

### 2.5. Metabolomic Approach

HPLC-MS/MS data were processed using MetaboAnalyst 6.0 on the HPLC-Q/TOF platform in negative mode. Spectra were converted to centWave-features, and peaks were aligned by quality-control-based retention–time correction (minFraction = 0.8) with contaminant removal. QC samples were used to correct retention differences and maintain data quality. The resulting peak matrix included extracts and fractions. Intensities were median normalized across samples, log^10^ transformed to reduce skewness and Pareto scale. Pattern–search correlation analysis was performed using the Score as the reference pattern and Pearson correlation as the distance measure. Methodology and parameter selection follow the MetaboAnalyst guidelines and Pang et al. [10].

### 2.6. Schistosomicidal Activity in Adult Worms

The life cycle of *S. mansoni* (Sambon, 1907) (Trematoda: Schistosomatidae) (BH strain—Belo Horizonte, MG, Brazil) was maintained in *Biomphalaria glabrata* (Say, 1818) (Gastropoda: Planorbidae) snails and female hamsters *Mesocricetus auratus* (Waterhouse, 1839) (Mammalia: Cricetidae) aged 4 weeks, freshly weaned, weighing 50–60 g, were housed in cages (30 × 20 × 13 cm) containing a sterile bed of wood shavings.

Each biological activity experiment used 4 female hamsters infected by subcutaneous injection of 300 cercariae, and six weeks later, *S. mansoni* adult worms were recovered by perfusion of the rodent’s portal and mesenteric system. The in vitro activity assay was performed according to a protocol established in our laboratory as previously described [6,7,9]. Shortly, adult worms were recovered through portal perfusion from hamsters 42 days after infection. Five coupled male and female worms were exposed to the test compounds; praziquantel was used as the positive control and DMSO as the negative control. Worms were maintained in 24-well culture plates at 37 °C and 5% CO_2_ and monitored after 2 h and then every 24 h thereafter for 96 h for motility, morphological alterations, and reproduction.

### 2.7. Score Construction

The anthelminthic activity was assessed by visual scoring of motility alterations. Scoring ranged from 0 to 150 with the higher values indicating major alterations. To ease interpretation, score values were classified and assigned to four categories according to the extent of effects: The category “Poor” scored 11–37 and includes worms with no or slight alterations on motility; “Regular” scored 38–80 displaying features intermediary between categories “Poor” and “Good”, which were worms with the no detectable movements and “No effects” scored 0–10 was attributed to groups with low % of worms with no or slight alterations on motility (Table 1).

## 3. Results and Discussion

In a previous study, we combined metabolomic approaches with analytical techniques to point out potential anthelminthic and molluscicide compounds in 45 crude extracts from 37 Brazilian seaweed species [7]. Multivariate analysis pointed towards 8 hits for molluscicidal activity and 33 hits for anthelminthic activity. After investigation based on published data, previous knowledge from chromatographic analysis and spectroscopic annotation of isolated compounds, triquinane alcohols, prenylated guaianes, dichotomanes, and xenianes were highlighted as potential active compounds. However, due to the high chemical complexity of the data, the metabolomic analysis was performed with taxonomically related groups that presented at least three active extracts and three inactive/relatively inactive extracts. Thus, the present study concentrated on refining the pre-analytical step of the protocol, aiming to generate fractions with reduced complexity and minimal overlap of metabolites.

The crude extracts were previously submitted to a liquid–liquid partition to the obtention of the dichloromethane phase. After comprehensive fractioning with solvents of different polarities, a wide range of metabolites with good resolution was obtained from the dichloromethane phase, contrasting with the complex data from the previous study. Non-polar to polar compounds were eluted subsequently according to solvent gradient, resulting in a clearer spectrum of chemical profile. Still, an additional fractionation step through HPLC-MS/MS was performed to the dereplication.

The crude extracts from the two selected macroalgae species *Laurencia aldingensis* and *L. dendroidea* were obtained with dichloromethane and methanol 1:1. Next, a liquid–liquid partition of the crude extract of *L. dendroidea* resulted in 1.840 mg (4.19%) of dichloromethane phase and 3.430 mg (7.80%) of hydromethanolic phase. The crude ex-tract of *L. aldingensis* resulted in 1.700 mg (4.69%) of dichloromethane phase and 2.720 mg (7.51%) of hydromethanolic phase.

The resulting dichloromethane phase was, therefore, submitted to a comprehensive fractioning of with solvents of different polarities, hexane, dichloromethane, ethyl acetate and methanol. For *L. dendroidea*, the hexane fraction resulted in 220 mg (12.94%), ethyl acetate fraction produced 420 mg (24.71%), dichloromethane fraction, 550 mg (32.35%) and the methanol fraction produced 550 mg (32.35%). For *L. aldingensis*, hexane fraction yielded 70 mg (4.11%), ethyl acetate, 290 mg (17.05%), dichloromethane fraction, 470 mg (27.64%), and methanolic fraction produced 240 mg (14.11%).

### 3.1. Biological Activity: Scoring Framework

The assessment of schistosomicidal activity has been based on phenotypical analysis [11,12,13]. However, despite being the gold standard in the evaluation of effects on schistosomes, subtle variations on biological effects are not detected. This gap was partially fulfilled with a score-based assessment [14,15,16,17], which still is not comprehensive enough, as the effects are scored at the end of the experiment within a narrow range and it does not allow to attribute a numerical value for a quantitative analysis. Still, to be inserted in a data matrix to a multivariate analysis, a numerical value must be attributed to biological effects. Usually, IC_50_ values are chosen to represent a biocide effect, but it is impossible to measure the wide range of subtle toxic effects at non-lethal doses. Therefore, in order to improve the score-based assessment, our group established a cumulative score, calculated throughout the experiment, resulting in a wider range (0–150) to measure the biological effects. Based on the obtained values, samples were classified in four categories, Poor (11–37), Regular (38–80), Good (81–150), and No Effects (0–10). Our results showed that *L. aldingensis* (Table 2) and *L. dendroidea* (Table 3) significantly affected viability and reproduction of exposed adult *S. mansoni* worms.

For *L. aldingensis*, the anthelminthic activities of hexane and dichloromethane fractions were classified as Good according to the score values—120 and 115, respectively, at 100 µg/mL. At 75 µg/mL, the dichloromethane activity was still classified as Good, but it decreased to Poor at the lowest concentration. On the other hand, the activities of methanol and aqueous fractions were classified as Poor, with score values of 10, 20 and 16; ethyl acetate was classified as no effect.

For *Laurencia dendroidea* (Table 2), the hexane fraction showed the highest activity, reaching a score of 139 with 100 µg/mL, being classified as Good. The dichloromethane fraction activity was classified as Regular, with score values of 80 and 40 with 100 µg/mL and 75 µg/mL, respectively, and Poor with score values of 37 at 50 µg/mL and 14 at 25 µg/mL. Etil acetate and methanol fractions activity was classified as Poor, with score values of 26 and 22; the hydromethanolic phase scored zero.

### 3.2. Biological Activity: Subfractions

As described in 2.3, dichloromethane fractions of both *Laurencia* species were submitted to the fractionation in HPLC at analytical scale. The fractions were collected according to the retention time and peak grouping, resulting in a total of 7 fractions for *L. dendroidea* and 7 fractions for *L. aldingensis*. From 84.2 mg of the dichloromethane fraction of *L. dendroidea*, a similar yield of all fractions was obtained, except for DCM—F4, which resulted in 30.5 mg (36.22%). From 71.5 mg of *L. aldingensis*, a similar yield was obtained for all the fractions.

The analysis of effects on viability and reproduction of *S. mansoni* showed that the subfractions of dichloromethane fraction were active against adult worms (Table 4 and Table 5). For *Laurencia aldingensis* (Table 4), subfractions DCM-F4 and DCM-F5 activity with 100 µg/mL and 75 µg/mL being classified as Good according to the score values; at 50 µg/mL and 25 µg/mL concentrations, the activity was scored as Poor or Regular (F4).

For *Laurencia dendroidea*, dichloromethane fractions DCM-F3, DCM-F4 and DCM-F6 induced 100% mortality. However, only DCM-F4 at 100 µg/mL was scored as Good, inducing 100% mortality after 24 h of exposure; at 75 µg/mL and 50 µg/mL, DCM-F4 was scored as regular.

### 3.3. Metabolite Profiling

**Figure 1:**
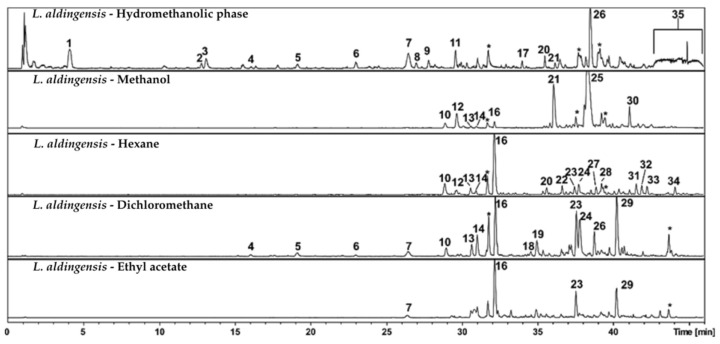
Chromatographic profile obtained by HPLC-MS in negative ionization mode of the polar partition (*L. aldingensis*—Hydromethanolic phase) and of the fractions obtained from the apolar partition—*L. aldingensis*—Hexane, *L. aldingensis*—Dichloromethane, *L. aldingensis*—Ethyl acetate, and *L. aldingensis*—Methanol. The numbers correspond to the peaks listed in Table 6. Peaks marked with (∗) representing contamination of the column.

The metabolites analysis of *Laurencia aldingensis* by HPLC-DAD-MS/MS (Figure 1, Table 6) reveals a complex chemical spectrum. Beginning with the compounds identified in peaks 4 to 7, a series of bromophenols was observed, including 5-bromo-3,4-dihydroxybenzaldehyde, and 3,5-dibromo-4-hydroxybenzoic acid—compounds known for their antioxidant properties [18,19]. The presence of these metabolites is characteristic of the *Laurencia* genus and suggests a defensive or signaling role against pathogens and herbivores in the marine environment [20,21]. These compounds are also of interest in the search for novel bioactive molecules with pharmacological potential.

Continuing the analysis, several peaks—namely 8, 9, 10, 12, 13, 14, 18, 19, 23, 24, and 29—could not be identified based on their molecular formulas or observed fragment ions. These compounds share the presence of both bromine and chlorine atoms in their structures and the absence of previously isolated substances matching their molecular formulas, suggesting that they may represent novel compounds not yet reported in scientific literature. For a specific subset—peaks 18, 19, 23, and 24—data indicated a possible structural analogy with halogenated sesquiterpenes of the aldigenin type, previously isolated from *Laurencia aldingensis* [22,23]. It is worth noting that the occurrence of halogenated structures is recurrent among red algal metabolites [24,25]. The possibility that these are new natural products highlights the relevance of future studies aimed at their isolation and full structural characterization, which could unveil new chemical entities and contribute to our understanding of marine biodiversity and its adaptive mechanisms.

The compound identified at peak 16, furocaespitanelactol—a member of the furocespitane class—is particularly intriguing, as it exhibits structural similarities to molecules previously reported in both algae and mollusks, suggesting the existence of a potentially conserved biosynthetic pathway [26,27,28]. Such pathways may be associated with symbiotic interactions or adaptations to specific ecological niches in the marine environment.

Hydroxylated fatty acids, such as those observed at peaks 20 and 22, represent a class of compounds that perform a variety of cellular functions, including membrane formation and signaling. These fatty acids are important structural components in algae and have implications for both algal physiology and its interactions with the surrounding environment [29,30]. Finally, peak 35 suggests a lobophorolide derivative, indicating the presence of long chain polyketides in *Laurencia aldingensis*. Polyketides are a class of secondary metabolites characterized by complex structures and a wide range of biological functions, ranging from chemical defense to the attraction of symbionts [31,32,33]. In summary, the data highlights the chemical complexity of *L. aldingensis*. The study of these compounds paves the way for a better understanding of the complex ecological interactions of this seaweed, as well as for the development of novel biotechnological and pharmaceutical resources. Mass spectrometry serves as a crucial tool for unraveling the chemical and biological complexity of these organisms, and each identified peak represents a potential starting point for future discoveries.

The data discussed above refers to the set of metabolites observed in the extracts of *L. aldingensis*. In the following section, we provide a more detailed discussion of the distribution of these compounds across the extracts and partitions presented in Figure 1, Table 6.

The hydromethanolic phase obtained through partitioning (Figure 1—*L. aldingensis*—Hydromethanolic phase), in which compound 1 and other highly polar compounds are prominent, as evidenced by their shorter retention times on the C18 column—indicating high affinity for water and the likely presence of polar functional groups. This fraction also contains polyketide 35, which was not detected in the other partitions, suggesting that the initial partitioning was effective in enriching this compound.

The hexane fraction was enriched in hydrophobic compounds, as evidenced by the prominent presence of peak 16 (furocaespitanelactol) and the more apolar aldingenin derivatives (peaks 23 and 29). In contrast, the dichloromethane (DCM) fraction exhibited a broader range of peaks, likely reflecting its ability to elute a wider spectrum of hydrophobic metabolites due to its relatively low polarity. Here, we observed a certain selectivity for furocaespitanelactol (peak 16) and aldingenin derivatives (peaks 18, 19, 23, 24, and 29), consistent with the expectation that DCM favors the extraction of compounds with higher hydrophobicity. This fraction also contained bromophenolic derivatives (peaks 4 to 7).

In the ethyl acetate fraction, compound 16 (furocaespitanelactol) and sesquiterpenoid structures related to the aldingenin skeleton, such as peaks 18 and 19, were prominent. These compounds suggest the presence of more complex and moderately polar structures that retain some solubility in this solvent. This indicates that these molecules possess structural features allowing moderate interactions with the silica stationary phase, favoring their elution with solvents of intermediate polarity.

**Figure 2:**
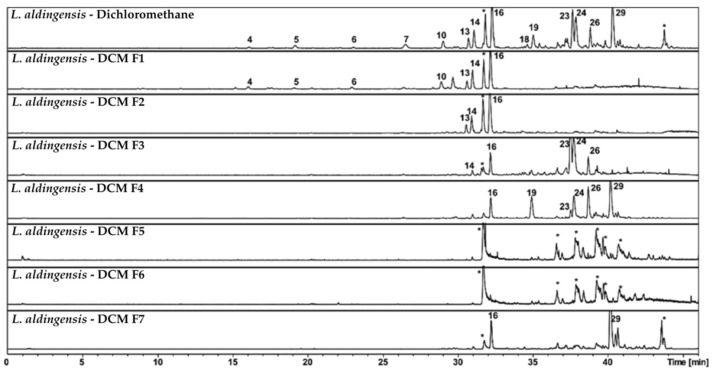
Chromatographic profile obtained by HPLC-MS in negative ionization mode of the *L. aldingensis*—Dichloromethane fraction and its subfractions F1–F7. The numbers correspond to the peaks listed in Table 6. Peaks marked with (∗) representing contamination of the column.

Finally, the methanolic fraction was enriched in more polar metabolites. Specifically, compounds 21 (C_26_H_40_O_11_) and 25 (C_28_H_44_O_11_), although not fully characterized, share similar molecular formulas, suggesting they belong to the same class of compounds. Figure 2 presents subfractions F1–F7, obtained through additional C8 column fractionation of the dichloromethane fraction (*L. aldingensis*—DCM). It highlights the capacity of reversed-phase chromatography to discriminate molecules based on polarity and their affinity for the hydrophobic stationary phase.

In the original *L. aldingensis*—Dichloromethane fraction, we observed a wide array of compounds, including peaks 4, 5, 6, 7, 13, 14, 16, 18, 19, 23, 24, and 29. These represent a mixture of bromophenols, terpenoids, and other apolar compounds, as indicated by their respective retention times.

With refinement through reversed-phase fractionation, a clear segregation of components began to emerge. Subfraction DCM—F1 retained many of the more polar compounds from the original mixture, as evidenced by peaks 4, 5, and 6. This suggests that these metabolites exhibit moderate interaction with the C8 stationary phase and are eluted with less apolar solvents in the mobile phase.

Subfractions DCM—F2 and DCM—F3 began to show a shift toward more apolar compounds, such as peak 16 (furocaespitanelactol), which appears consistently across multiple fractions. The persistent presence of this compound suggests a significant affinity for the C18 stationary phase, requiring a stronger elution gradient to be displaced from the column, or alternatively, that it is present at high concentration, saturating the system and appearing in several subfractions.

Moving to subfractions DCM—F4 through DCM—F7, we observed a progressive decrease in compound diversity. In particular, DCM—F5 and DCM—F6 were predominantly composed of column related contaminants; however, it is also possible that these subfractions contain non-ionizable compounds that were not detected under the current MS conditions. Peak 16 remained dominant, and peak 29 occurred in DCM—F7, suggesting that this compound requires more specific elution conditions, possibly due to its higher hydrophobicity or larger molecular size.

These results illustrate the principle that reversed-phase C8 chromatography can be fine-tuned to separate compounds based on subtle differences in polarity and solubility. With appropriate optimization of the elution gradient, compounds such as furocaespitanelactol and aldingenin derivatives—which may possess biological or pharmaceutical relevance—can be effectively isolated for further analysis.

**Figure 3:**
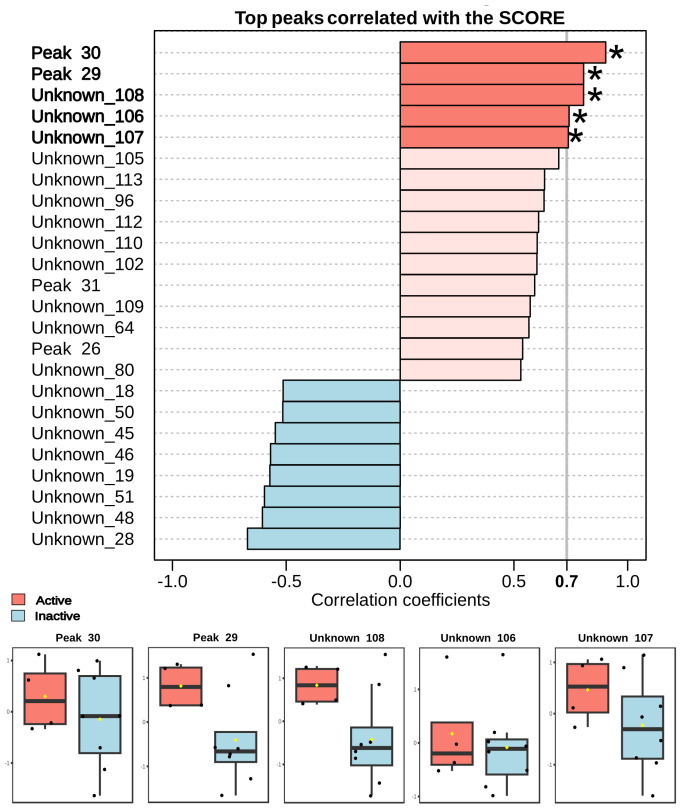
Pearson correlation analysis of the 25 hits selected in the LC-MS/MS analysis of *Laurencia aldingensis* fractions and schistosomicidal activity in *Schistosoma mansoni*. Black spots corresponding to the samples and yellow spots to the means. Peaks marked with (∗) representing hits.

The Pearson correlation analysis (Figure 3) clearly highlights specific chemical compounds highly associated with the observed anthelminthic activity in fractions of *Laurencia aldingensis*. Notably, compounds annotated as Peak 30, Peak 29, Unknown_108, Unknown_106, and Unknown_107 demonstrated strong positive correlations (correlation coefficient > 0.7). Boxplots provided below the correlation graph further emphasize that these metabolites exhibit substantially higher intensities in active fractions compared to inactive ones, reinforcing their potential key role in the observed biological activity.

Furthermore, the unknown metabolites (Unknown_106, Unknown_107, and Unknown_108) also display high correlation, likely representative of novel bioactive compounds not previously reported. Future research focusing on the isolation, characterization, and biological evaluation of these metabolites is critical and could significantly advance therapeutic strategies against schistosomiasis. Conversely, metabolites showing negative correlations (e.g., Unknown_28, Unknown_48, Unknown_51) may serve as negative chemical markers to streamline and optimize bioactivity guided purification protocols.

**Figure 4:**
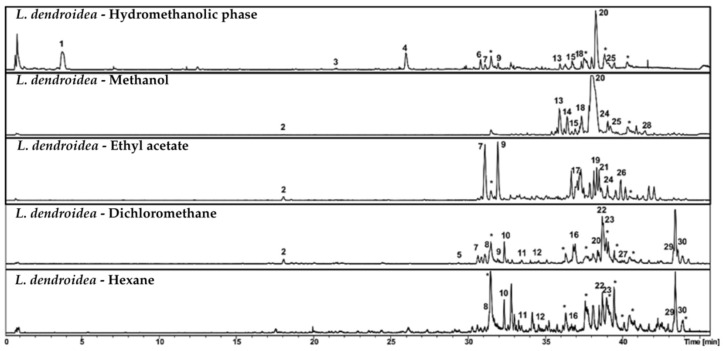
Chromatographic profile by HPLC-DAD-MS in negative ionization mode of the polar partition (*L. dendroidea*—Hydromethanolic phase) and the fractions obtained from the apolar partition: *L. dendroidea*—Hexane, *L. dendroidea*—Dichloromethane, *L. dendroidea*—Ethyl acetate and *L. dendroidea*—Methanol. The numbers correspond to the peaks listed in Table 7. Peaks marked with (∗) representing contamination of the column.

**Figure 5:**
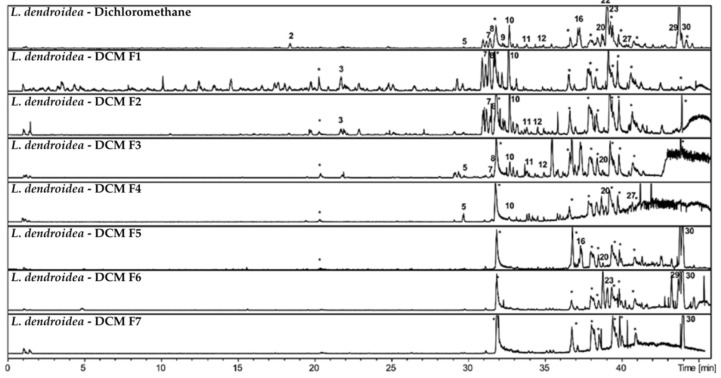
Chromatographic profile by HPLC-DAD-MS in negative ionization mode of the *L. dendroidea* Dichloromethane fraction and its subfractions F1—F7. The numbers correspond to the peaks listed in Table 7. Peaks marked with (∗) representing contamination of the column.

Chromatographic analysis by HPLC-DAD-MS/MS revealed a high chemical diversity in fractions from *Laurencia dendroidea*, comparable to that observed in *L. aldingensis* (Figure 4 and Figure 5). The dichloromethane fraction was selected for subfractionation due to its remarkable activity and its shared compounds with other fractions.

Major compounds in this fraction included oxygenated sesquiterpenes, fatty acid derivatives, and halogenated metabolites (Table 7). Peaks 8 and 10 were annotated as laurecomin D and hydroxy laurecomin D, while Peaks 2, 7, 8, and 11 presented fragmentation patterns and molecular formulas consistent with yet undescribed sesquiterpenoid derivatives.

Among fatty acid derivatives, Peak 29 was identified as hydroxy eicosanoic acid [34]. Peaks 22 and 23 were annotated as penicitide A isomers, a polyketide previously isolated from *Penicillium chrysogenum*, an endophytic fungus associated with *Laurencia* species [35]. Peak 30 was identified as the marine-derived steroid muristeroid G [36].

Chromatographic profiles of subfractions F1–F7 (Figure 4), obtained from the dichloromethane fraction via reverse-phase C8 column, showed effective separation by polarity. More polar compounds were concentrated in the initial subfractions, while less polar compounds were found in later ones. Several peaks absent or at low intensity in the original fraction became enriched in the subfractions, highlighting the efficiency of the chromatographic process.

**Figure 6:**
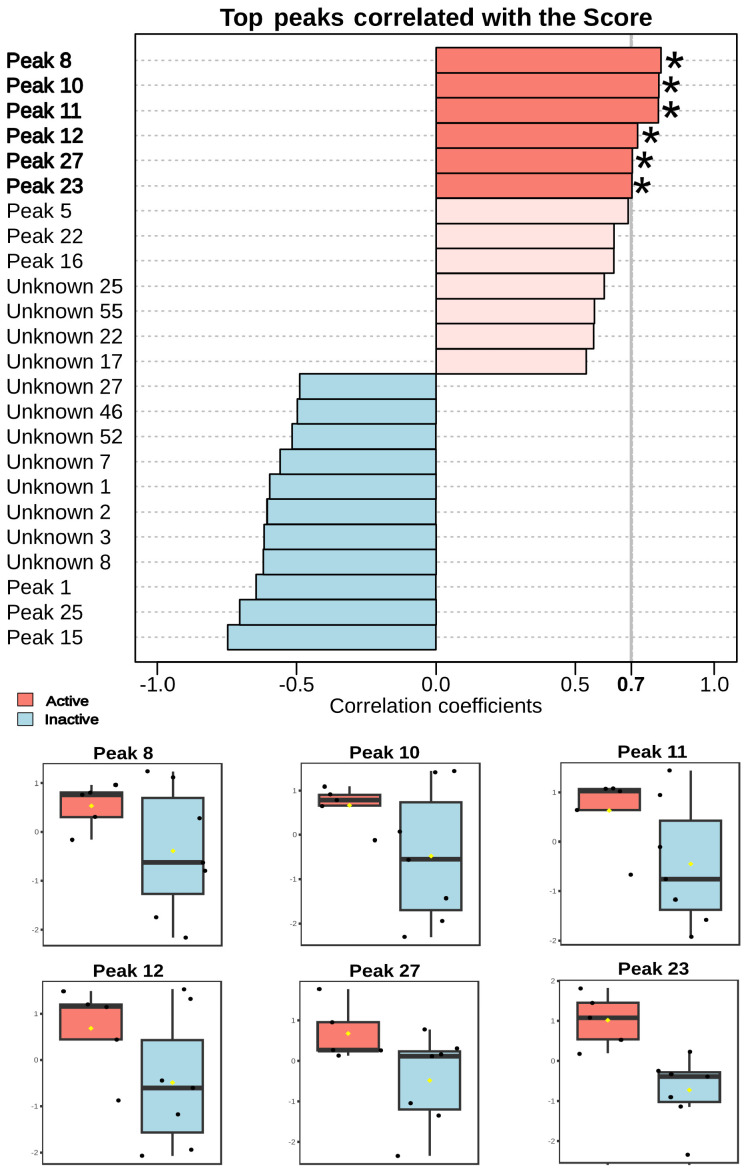
Pearson correlation analysis of the 25 hits selected in the LC-MS/MS analysis of *Laurencia dendroidea* fractions and schistosomicidal activity in *Schistosoma mansoni*. Black spots corresponding to the samples and yellow spots to the means. Peaks marked with (∗) representing hits.

Statistical analysis revealed that several LC-MS peaks showed a significant positive correlation (*p* < 0.05) with biological activity against *Schistosoma mansoni* (Figure 6).

One of most notable was penicitide A (23, r = 0.744; *p* = 0.0056), which was exclusive to active fractions. Other significantly correlated peaks included 11 (r = 0.799; *p* = 0.0018), 12 (r = 0.723; *p* = 0.0078), and 27 (r = 0.704; *p* = 0.011), all being halogenated compounds. Additionally, laurecoin D (8, r = 0.808; *p* = 0.0015) and hydroxy laurecomin D (10, r = 0.800; *p* = 0.0018) also showed strong correlations with biological activity. Both are halogenated sesquiterpenes previously reported in *Laurencia composita* [37].

The strong correlation between schistosomicidal activity and penicitide A (23) suggests a direct involvement of these compounds in the biological effect of *Laurencia dendroidea*. Penicitide A belongs to a class of polyketides previously reported for antimicrobial properties, although its activity against *Schistosoma mansoni* has not yet been described [35]. The exclusive presence of 23 in active fractions supports its potential as a bioactive lead compound.

Peaks 11, 12, and 27, annotated as halogenated but uncharacterized molecules, also showed positive correlations with biological activity. The presence of bromine and chlorine atoms in their structures is consistent with typical metabolites from the *Laurencia* genus, which are known for their biochemical diversity and broad pharmacological potential. Halogenated marine natural products have been associated with enzyme inhibition and disruption of membrane integrity in helminths [38].

Sesquiterpenes such as laurecomin D and its hydroxylated derivative (10 and 8) also showed high correlation coefficients. These compounds have been previously described in *Laurencia composita* with antimicrobial and cytotoxic activities [37]. While their role in schistosomicidal activity remains to be confirmed, their presence in active fractions suggests potential involvement (Figure 6).

The observed diversity of compounds and their distribution across fractions and subfractions suggests that the schistosomicidal effect may result from synergistic interactions among different classes of metabolites, particularly halogenated sesquiterpenes and fatty acid derivatives. This pattern is consistent with findings in *L. aldingensis*, where bioactivity has also been linked to chemical synergy.

Overall, these results underscore the pharmacological potential of *Laurencia dendroidea* as a source of novel schistosomicidal agents. Further work is needed to isolate and structurally characterize the peaks most strongly correlated with activity—especially the penicitide A—and to evaluate their bioactivity through in vitro and in vivo assays. The elucidation of yet uncharacterized halogenated metabolites (e.g., 11, 12, and 27) may also lead to the discovery of new marine-derived bioactive scaffolds.

## 4. Conclusions

In conclusion, this study successfully demonstrates the effectiveness of a refined metabolomics-based approach in identifying promising bioactive compounds from *Laurencia aldingensis* and *L. dendroidea* with significant schistosomicidal activities. The optimized extraction and fractionation procedures generated samples of reduced chemical complexity and minimal metabolite overlap, enabling precise and robust chemical characterization via high-resolution mass spectrometry (HRMS). The observed chemical diversity and the distinct distribution patterns across fractions and subfractions strongly indicate that the anthelminthic activity may arise from synergistic interactions among multiple classes of metabolites, particularly halogenated sesquiterpenes and fatty acid derivatives.

The significant correlations identified through statistical analysis highlighted several metabolites—such as bromophenols, halogenated sesquiterpenes, and potentially novel bioactive structures—as primary candidates underlying the observed antiparasitic effects. *Laurencia dendroidea*, in particular, emerges as a valuable source of novel schistosomicidal agents. Future research should prioritize the isolation, structural elucidation, and comprehensive pharmacological evaluation of these highly correlated compounds, specifically focusing on promising candidates such as penicitide A. Additionally, the characterization of previously unidentified halogenated metabolites (e.g., peaks 11, 12, and 27) could provide novel marine-derived chemical scaffolds with significant therapeutic potential. Overall, advancing the understanding of these marine macroalgal metabolites not only contributes to marine biodiversity knowledge and chemical ecology but also represents an essential step toward developing innovative treatments for schistosomiasis.

## Figures and Tables

**Table 1:** Parameters used to construct the score.

Parameter	Classification	Score
Motility of each helminth	Normal	0
	Slightly reduced	1
	Significantly reduced	2
	No movements	3

Score = ∑ (M♀p + M♂p). M = motility score, p = time of exposure, ♀ = female; ♂ = male.

**Table 2:** Schistosomicidal effect of marine macroalgae *Laurencia aldingensis* Y. Saito and Womersley 1974 fractions on *Schistosoma mansoni* worms.

Concentration (µg/mL)	Fraction	Score	Classification
100 µg/mL	Hydromethanolic phase	16	Poor
	Methanol	20	Poor
	Ethyl acetate	10	No effect
	Dichloromethane	120	Good
	Hexane	115	Good
75 µg/mL	Dichloromethane	104	Good
50 µg/mL	Dichloromethane	50	Regular
25 µg/mL	Dichloromethane	20	Poor
Positive control	Praziquantel 1.5 µg/mL	123	Good
Negative control	DMSO 1.5% µg/mL	0	No effect

Score track: No effect (0–10); Poor (11–37); Regular (38–80); Good (81–150).

**Table 3:** Schistosomicidal effect of marine macroalgae *Laurencia dendroidea* J. Agardh 1852 fractions on *Schistosoma mansoni* worms.

Concentration (µg/mL)	Fraction	Score	Classification
100 µg/mL	Hydromethanolic phase	0	No effect
	Methanol	22	Poor
	Ethyl acetate	26	Poor
	Dichloromethane	80	Regular
	Hexane	139	Good
75 µg/mL	Dichloromethane	40	Regular
50 µg/mL	Dichloromethane	37	Poor
25 µg/mL	Dichloromethane	14	Poor
Positive control	Praziquantel 1.5 µg/mL	123	Good
Negative control	DMSO 1.5% µg/mL	0	No effect

Score track: No effect (0–10); Poor (11–37); Regular (38–80); Good (81–150).

**Table 4:** Schistosomicidal effect of marine macroalgae *Laurencia aldingensis* Y. Saito and Womersley 1974 subfractions on *Schistosoma mansoni* worms.

Concentration (µg/mL)	Subfraction	Score	Classification
100 µg/mL	DCM—F1	0	No effect
	DCM—F2	0	No effect
	DCM—F3	0	No effect
	DCM—F4	130	Good
	DCM—F5	120	Good
	DCM—F6	16	Poor
	DCM—F7	16	Poor
75 µg/mL	DCM—F4	120	Good
	DCM—F5	94	Good
50 µg/mL	DCM—F4	64	Regular
	DCM—F5	28	Poor
25 µg/mL	DCM—F4	0	No effect
	DCM—F5	0	No effect
Positive control	Praziquantel 1.5 µg/mL	120	Good
Negative control	DMSO 1.5% µg/mL	0	No effect

Score track: No effect (0–10); Poor (11–37); Regular (38–80); Good (81–150).

**Table 5:** Schistosomicidal effect of marine macroalgae *Laurencia dendroidea* J. Agardh 1852 subfractions on *Schistosoma mansoni* worms.

Concentration (µg/mL)	Subfraction	Score	Classification
100 µg/mL	DCM—F1	20	Poor
	DCM—F2	25	Poor
	DCM—F3	48	Regular
	DCM—F4	120	Good
	DCM—F5	12	Poor
	DCM—F6	79	Regular
	DCM—F7	0	No effect
75 µg/mL	DCM—F3	55	Regular
	DCM—F4	66	Regular
	DCM—F6	24	Poor
50 µg/mL	DCM—F3	26	Poor
	DCM—F4	46	Regular
	DCM—F6	16	Poor
25 µg/mL	DCM—F3	4	No effect
	DCM—F4	26	Poor
	DCM—F6	20	Poor
Positive control	Praziquantel 1.5 µg/mL	120	Good
Negative control	DMSO 1.5% µg/mL	0	No effect

Score track: No effect (0–10); Poor (11–37); Regular (38–80); Good (81–150).

**Table 6:** Chemical profile of *Laurencia aldingensis* Y. Saito and Womersley 1974 determined by HPLC-DAD-MS/MS.

Peak	TR min.	UV	*m*/*z*	Fragmentation	Formula	Annotation
1	4.1		303.1926	216(C_10_H_18_NO_4_)	C_14_H_28_N_2_O_5_	unknown
2	12.9		241.1215	197(C_10_H_17_N_2_O_2_)	C_11_H_18_N_2_O_4_	unknown
3	13.2		189.0801	---	C_8_H_14_O_5_	unknown
4	16.2	255/285	214.9379	---	C_7_H_5_BrO_3_	5-Bromo-3,4-dihydroxybenzaldehyde
5	19.2	276	198.9419	---	C_7_H_5_BrO_2_	3-Bromo-4-hydroxybenzaldehyde
6	23.1	260	292.8456	248(C_6_H_3_Br_2_O)	C_7_H_4_Br_2_O_3_	3,5-Dibromo-4-hydroxybenzoic acid
7	26.5	277	276.8443	---	C_7_H_4_Br_2_O_2_	3,5-Dibromo-4-hydroxybenzaldehyde
8	27.1	---	427.9923	394(C_17_H_15_BrNO_5_). 348(C_17_H_15_ClNO_5_), 312(C_17_H_14_NO_5_)	C_17_H_17_BrClNO_5_	unknown
9	27.9	---	411.9981	378(C_17_H_15_BrNO_4_). 332(C_17_H_15_ClNO_4_), 296(C_17_H_14_NO_4_)	C_17_H_17_BrClNO_4_	unknown
10	29.0	---	354.9955	275(C_12_H_16_ClO_5_), 239(C_12_H_15_O_5_)	C_12_H_18_BrClO_5_	unknown
11	29.6	---	411.9973	378(C_17_H_15_BrNO_4_). 332(C_17_H_15_ClNO_4_), 296(C_17_H_14_NO_4_). 209 (C_11_H_13_O_4_)	C_17_H_17_BrClNO_4_	unknown
12	29.7	---	354.9951	275(C_12_H_16_ClO_5_), 239(C_12_H_15_O_5_)	C_12_H_18_BrClO_5_	unknown
13	30.6	---	252.9637	---	C_8_H_12_BrClO_2_	unknown
14	31.1	---	339.0016	223(C_12_H_15_O_4_)	C_12_H_18_BrClO_4_	unknown
15	31.2	---	223.1044	---	C_12_H_16_O_4_	Terpene Derivative
16	32.2	---	320.9906	---	C_12_H_16_BrClO_3_	Furocaespitanelactol
17	34.1	---	385.2052		C_23_H_30_O_5_	unknown
18	34.6		460.9659	---	C_15_H_25_Br_2_ClO_4_	Aldingenin Derivative
19	35.0	---	444.9751	249(C_14_H_17_O_4_)	C_15_H_25_Br_2_ClO_3_	Aldingenin Derivative
20	35.5	---	287.2244	---	C_16_H_32_O_4_	Dihydroxypalmitic acid
21	36.2	---	527.2520	---	C_26_H_40_O_11_	unknown
22	36.7	---	269.2137	---	C_16_H_30_O_3_	Hydroxypalmitic acid
23	37.7	---	488.9701	---	C_16_H_25_Br_2_ClO_5_	Aldingenin Derivative
24	37.8	---	490.9866	---	C_16_H_27_Br_2_ClO_5_	Aldingenin Derivative
25	38.4	---	555.2886	225(C_9_H_5_O_7_)	C_28_H_44_O_11_	unknown
26	38.8	---	533.0068	269(C_16_H_29_O_3_)	C_22_H_29_Br_2_ClO_3_	unknown
27	38.8	---	537.3304	255(C_16_H_31_O_2_)	C_26_H_50_O_11_	unknown
28	39.2	---	537.3323	255(C_16_H_31_O_2_)	C_26_H_50_O_11_	unknown
29	40.3	---	474.9910	269(C_16_H_29_O_3_), 213(C_13_H_25_O_2_)	C_16_H_27_Br_2_ClO_4_	Aldingenin Derivative
30	41.1	---	271.2286	255(C_15_H_29_O)	C_16_H_32_O_3_	unknown
31	41.5	---	340.2861	310(C_19_H_36_NO_2_), 268 (C_17_H_34_NO)	C_20_H_39_NO_3_	unknown
32	42.0	---	384.3129	255(C_16_H_31_O_2_)	C_22_H_43_NO_4_	unknown
33	42.3	---	384.3135	255(C_16_H_31_O_2_)	C_22_H_43_NO_4_	unknown
34	44.1	---	309.2428	265(C_18_H_34_O)	C_19_H_34_O_3_	Hydroxy fatty acids derivative
35	44.5	---	765.4752	---	C_42_H_70_O_12_	putative Lobophorolide derivate

**Table 7:** Chemical profile of *Laurencia dendroidea* J. Agardh 1852 determined by HPLC-DAD-MS/MS.

Peak	TR min.	*m*/*z*	Fragmentation	Formula	Annotation
1	4.17	303.1925	244, 216, 170	C_15_H_24_N_6_O	Unknown
2	18.38	283.1548	-	C_15_H_24_O_5_	Unknown
3	21.77	283.1549	211	C_11_H_20_N_6_O_3_	Unknown
4	26.41	209.0853	209	C_9_H_14_N_4_S	Unknown
5	29.67	215.1282	197, 169	C_11_H_20_O_4_	Unknown
6	31.11	223.1010	223	C_10_H_16_N_4_S	Unknown
7	31.43	267.1600	267, 249, 223	C_15_H_24_O_4_	2,3,5,6,7,7a-Hexahydro-2,7-dihydroxy-3-(hydroxymethyl)-1,1,3,5-tetramethyl-1H-indene-4-carboxaldehyde
8	31.63	393.0106	397, 395, 393, 315, 313, 277, 233, 221	C_15_H_20_BrClO_5_	Hidroxy laurecomin D
9	32.26	267.1594	267, 223, 180	C_15_H_24_O_4_	Trichocarotin G
10	32.66	379.0311	383, 381, 379, 299, 263, 221, 219	C_15_H_22_BrClO_4_	Laurecomin D
11	33.76	349.0211	251, 193	C_14_H_20_BrClO_3_	Unknown
12	34.85	347.0049	-	C_14_H_18_BrClO_3_	Unknown
13	36.28	527.2537	225	C_23_H_44_O_11_S	2-Hydroxy-3-[(1-oxotetradecyl)oxy]propyl 6-deoxy-6-sulfo-α-D-glucopyranoside
14	36.78	601.2686	225	C_29_H_46_O_11_S	Unknown
15	37.04	287.2229	241, 223	C_16_H_32_O_4_	Unknown
16	37.08	285.2071	267, 223, 183	C_16_H_30_O_4_	Unknown
17	37.36	317.2125	317, 299, 255, 195, 167	C_20_H_30_O_3_	Unknown
18	37.63	653.3758	397	C_31_H_58_O_14_	Unknown
19	38.54	319.2293	319, 301, 257	C_20_H_32_O_3_	Hydroxyeicosatetraenoic acid
20	38.54	555.2853	225	C_25_H_48_O_11_S	3-Hydroxy-2-[(1-oxohexadecyl)oxy]propyl 6-deoxy-6-sulfo-β-D-galactopyranoside
21	38.55	319.2281	-	C_20_H_32_O_3_	Hydroxyeicosatetraenoic acid
22	39.04	313.2384	313, 295, 225	C_18_H_34_O_4_	Penicitide A isomer
23	39.06	313.2384	313, 295	C_18_H_34_O_4_	Penicitide A isomer
24	39.34	491.3218	491, 255	C_25_H_48_O_9_	2-Hydroxy-3-[(1-oxohexadecyl)oxy]propyl β-D-galactopyranoside
25	39.59	483.2735	-	C_24_H_40_N_2_O_8_	Unknown
26	40.13	400.3064	400	C_22_H_43_NO_5_	Unknown
27	40.20	474.9901	-	C_16_H_27_Br_2_ClO_4_	Unknown
28	41.66	297.2442	297, 251	C_18_H_34_O_3_	Unknown
29	43.53	327.2906	327, 255	C_20_H_40_O_3_	Unknown
30	43.86	433.3328	433	C_27_H_46_O_4_	Muristeroid G

## Data Availability

The original contributions presented in the study are included in the article/Supplementary Material; further inquiries can be directed to the corresponding author.

## Supplementary Materials

The following are available online at https://www.mdpi.com/article/10.3390/pharmaceutics17101294/s1, S1: Schistosomicidal effect of marine macroalgae *Laurencia aldingensis* Y. Saito and Womersley 1974 fractions on *Schistosoma mansoni* worms. S2: Schistosomicidal effect of marine macroalgae *Laurencia dendroidea* J. Agardh 1852 fractions on *Schistosoma mansoni* worms. S3: Schistosomicidal effect of marine macroalgae *Laurencia aldingensis* Y. Saito and Womersley 1974 subfractions on *Schistosoma mansoni* worms. S4: Schistosomicidal effect of marine macroalgae *Laurencia dendroidea* J. Agardh 1852 subfractions on *Schistosoma mansoni* worms. S5: List of entries used in the statistical correlation analyses between compounds detected in the HPLC-MS chromatograms (negative ion mode) and the schistosomicidal activity score. S6: List of entries used in the statistical correlation analyses between compounds detected in the HPLC-MS chromatograms (negative ion mode) and the schistosomicidal activity score. S7: Schistosomicidal Activity in Adult Worms: Animal care and monitoring.
