# Supplementary material for: Use of Metabolomics Approach in the Discovery of Active Compounds from Macroalgae Laurencia Species Against Schistosomiasis
Source: Pharmaceutics. 2025 Oct 2;17(10):1294. doi: 10.3390/pharmaceutics17101294 (PMC12567142; doi:10.3390/pharmaceutics17101294)
Supplement: Supplementary file 1 [file pharmaceutics-17-01294-s001.zip › pharmaceutics-3763482-supplementary.pdf]

# Supplementary Materials: Use of Metabolomics Approach in the Discovery of Active Compounds from Macroalgae *Laurencia* Species Against Schistosomiasis

**Amanda Beatriz Silva Soares [1,2,3], Patricia Aoki Miyasato [1], Rafaela Paula de Freitas [1], Adolfo Luis Almeida Maleski [4], Daniel Carvalho Pimenta [5,6], Pio Colepicolo [2], Erika Mattos Stein [2], Arthur Ladeira Macedo [7], Carlos Alexandre Carollo [7] and Eliana Nakano [1,3,*]**

**S1:** Schistosomicidal effect of marine macroalgae *Laurencia aldingensis* Y. Saito and Womersley 1974 fractions on *Schistosoma mansoni* worms.

| Fractions | Mortality (%) | | | Eggs (average) |
|---|---|---|---|---|
| | Females | Males | Total | |
| Hidrometanolic phase | | | | |
| 100µg/mL | 0 | 0 | 0 | 56.4 |
| Hexane | | | | |
| 100µg/mL | 100 | 100 | 100 | 0 |
| Dichloromethane | | | | |
| 100µg/mL | 100 | 100 | 100 | 0 |
| 75µg/mL | 0 | 0 | 0 | 0 |
| 50µg/mL | 0 | 0 | 0 | 26.8 |
| 25µg/mL | 0 | 0 | 0 | 67.4 |
| Ethyl acetate | | | | |
| 100µg/mL | 0 | 0 | 0 | 44.6 |
| 75µg/mL | 0 | 0 | 0 | 71.6 |
| 50µg/mL | 0 | 0 | 0 | 95.2 |
| 25µg/mL | 0 | 0 | 0 | 106.8 |
| Methanol | | | | |
| 100µg/mL | 0 | 0 | 0 | 74.6 |
| 75µg/mL | 0 | 0 | 0 | 87.4 |
| 50µg/mL | 0 | 0 | 0 | 100.0 |
| 25µg/mL | 0 | 0 | 0 | 103.6 |
| Controls | | | | |
| Positive* | 0 | 100 | 50 | 0 |
| Negative** | 0 | 0 | 0 | 112.4 |

Legend: *Praziquantel 1.5 µg/mL; **RPMI media with DMSO 1.5% µg/mL. Five pairs of adult worms were incubated in wells with the fractions.

**S2:** Schistosomicidal effect of marine macroalgae *Laurencia dendroidea* J. Agardh 1852 fractions on *Schistosoma mansoni* worms.

| Fractions | Mortality (%) | | | Eggs (average) |
|---|---|---|---|---|
| | Females | Males | Total | |
| Hidrometanolic phase | | | | |
| 100μg/mL | 0 | 0 | 0 | 22.0 |
| Hexane | | | | |
| 100μg/mL | 100 | 100 | 100 | 0 |
| Dichloromethane | | | | |
| 100μg/mL | 100 | 100 | 100 | 0 |
| 75μg/mL | 100 | 100 | 100 | 0 |
| 50μg/mL | 0 | 0 | 0 | 0 |
| 25μg/mL | 0 | 0 | 0 | 20.2 |
| Ethyl acetate | | | | |
| 100μg/mL | 0 | 0 | 0 | 3.0 |
| 75μg/mL | 0 | 0 | 0 | 24.4 |
| 50μg/mL | 0 | 0 | 0 | 33.8 |
| 25μg/mL | 0 | 0 | 0 | 53.4 |
| Methanol | | | | |
| 100μg/mL | 0 | 0 | 0 | 123.4 |
| 75μg/mL | 0 | 0 | 0 | 111.0 |
| 50μg/mL | 0 | 0 | 0 | 122.2 |
| 25μg/mL | 0 | 0 | 0 | 106.8 |
| Controls | | | | |
| Positive* | 0 | 100 | 50 | 0 |
| Negative** | 0 | 0 | 0 | 129.2 |

Legend: *Praziquantel 1.5 μg/mL; **RPMI media with DMSO 1.5% μg/mL. Five pairs of adult worms were incubated in wells with the fractions.

**S3:** Schistosomicidal effect of marine macroalgae *Laurencia aldingensis* Y. Saito and Womersley 1974 subfractions on *Schistosoma mansoni* worms.

| Subfractions | Mortality (%) | | | Eggs (average) |
|---|---|---|---|---|
| | Females | Males | Total | |
| DCM – F1 | | | | |
| 100µg/mL | 0 | 0 | 0 | 91.2 |
| 75µg/mL | 0 | 0 | 0 | 89.6 |
| 50µg/mL | 0 | 0 | 0 | 120.6 |
| 25µg/mL | 0 | 0 | 0 | 122.0 |
| DCM – F2 | | | | |
| 100µg/mL | 0 | 20 | 10 | 8.2 |
| DCM – F3 | | | | |
| 100µg/mL | 0 | 0 | 0 | 0 |
| 75µg/mL | 0 | 0 | 0 | 0 |
| 50µg/mL | 0 | 0 | 0 | 38.0 |
| 25µg/mL | 0 | 0 | 0 | 84.2 |
| DCM – F4 | | | | |
| 100µg/mL | 100 | 100 | 100 | 0 |
| 75µg/mL | 60 | 60 | 60 | 0 |
| 50µg/mL | 0 | 0 | 0 | 0 |
| 25µg/mL | 0 | 0 | 0 | 26.8 |
| DCM – F5 | | | | |
| 100µg/mL | 0 | 80 | 40 | 44.0 |
| DCM – F6 | | | | |
| 100µg/mL | 100 | 100 | 100 | 11.0 |
| 75µg/mL | 0 | 0 | 0 | 36.2 |
| 50µg/mL | 0 | 0 | 0 | 73.6 |
| 25µg/mL | 0 | 0 | 0 | 114.4 |
| DCM – F7 | | | | |
| 100µg/mL | 0 | 0 | 0 | 103.8 |
| Controls | | | | |
| Positive* | 0 | 100 | 50 | 0 |
| Negative** | 0 | 0 | 0 | 116.4 |

Legend: *Praziquantel 1.5 µg/mL; **RPMI media with DMSO 1.5% µg/mL. Five pairs of adult worms were incubated in wells with the subfractions.

**S4:** Schistosomicidal effect of marine macroalgae *Laurencia dendroidea* J. Agardh 1852 subfractions on *Schistosoma mansoni* worms.

| Subfractions | Mortality (%) | | | Eggs (average) |
|---|---|---|---|---|
| | Females | Males | Total | |
| DCM – F1 | | | | |
| 100µg/mL | 0 | 0 | 0 | 105.0 |
| 75µg/mL | 0 | 0 | 0 | 110.4 |
| 50µg/mL | 0 | 0 | 0 | 112.0 |
| 25µg/mL | 0 | 0 | 0 | 109.8 |
| DCM – F2 | | | | |
| 100µg/mL | 0 | 0 | 0 | 81.0 |
| 75µg/mL | 0 | 0 | 0 | 90.4 |
| 50µg/mL | 0 | 0 | 0 | 121.6 |
| 25µg/mL | 0 | 0 | 0 | 115.2 |
| DCM – F3 | | | | |
| 100µg/mL | 0 | 0 | 0 | 0 |
| 75µg/mL | 0 | 0 | 0 | 0 |
| 50µg/mL | 0 | 0 | 0 | 5.8 |
| 25µg/mL | 0 | 0 | 0 | 59.2 |
| DCM – F4 | | | | |
| 100µg/mL | 100 | 100 | 100 | 0 |
| 75µg/mL | 100 | 100 | 100 | 0 |
| 50µg/mL | 0 | 0 | 0 | 0 |
| 25µg/mL | 0 | 0 | 0 | 0 |
| DCM – F5 | | | | |
| 100µg/mL | 100 | 100 | 100 | 0 |
| 75µg/mL | 80 | 80 | 80 | 0 |
| 50µg/mL | 0 | 0 | 0 | 0 |
| 25µg/mL | 0 | 0 | 0 | 32.2 |
| DCM – F6 | | | | |
| 100µg/mL | 0 | 0 | 0 | 49.4 |
| DCM – F7 | | | | |
| 100µg/mL | 0 | 0 | 0 | 91.2 |
| Controls | | | | |
| Positive* | 40 | 100 | 70 | 0 |
| Negative** | 0 | 0 | 0 | 116.4 |

Legend: *Praziquantel 1.5 µg/mL; **RPMI media with DMSO 1.5% µg/mL. Five pairs of adult worms were incubated in wells with the subfractions.

**S5:** List of entries used in the statistical correlation analyses between compounds detected in the HPLC-MS chromatograms (negative ion mode) and the schistosomicidal activity score.

| Entry | Rt | *m/z* | La-HeX | La-DCM | La-AcOEt | La-MEOH | La-H2O | La-F1 | La-F2 | La-F3 | La-F4 | La-F5 | La-F6 | La-F7 |
|---|---|---|---|---|---|---|---|---|---|---|---|---|---|---|
| | | | Active | Active | Inactive | Inactive | Inactive | Inactive | Inactive | Inactive | Active | Active | Inactive | Inactive |
| Unknown_1 | 1.3 | 152.0403 | 210.5 | 122.2 | 14.4 | 3555.6 | 223721.9 | 477.3 | 114.7 | 170.9 | 39.9 | 82.2 | 109.4 | 30.4 |
| Unknown_2 | 1.3 | 289.0701 | 55.9 | 27.9 | 103.5 | 1435.4 | 196325.9 | 59.0 | 12.5 | 24.7 | 15.9 | 0.2 | 8.7 | 13.9 |
| Unknown_3 | 1.76 | 243.0635 | 65.2 | 65.2 | 260.7 | 372.5 | 175105.0 | 85.0 | 38.5 | 819.6 | 53.0 | 15.2 | 0.1 | 96.5 |
| Unknown_4 | 2.42 | 267.0741 | 32.7 | 0.7 | 82.6 | 462.2 | 135629.5 | 38.4 | 15.0 | 54.6 | 24.6 | 0.4 | 0.8 | 7.0 |
| Unknown_5 | 4.14 | 216.1243 | 42.1 | 53.9 | 22.9 | 1094.3 | 325235.3 | 34.5 | 8.9 | 0.8 | 12.9 | 0.4 | 0.6 | 0.8 |
| Peak_1 | 4.15 | 303.1931 | 0.5 | 0.4 | 0.1 | 50.4 | 101461.7 | 0.3 | 0.2 | 22.4 | 0.6 | 0.5 | 0.2 | 15.8 |
| Peak_2 | 12.82 | 241.1193 | 33.9 | 104.6 | 115.4 | 193.4 | 62129.4 | 291.5 | 319.5 | 101.2 | 49.2 | 0.4 | 16.0 | 43.4 |
| Peak_3 | 13.14 | 189.0768 | 42.2 | 30.9 | 159.0 | 201.1 | 160868.9 | 76.1 | 26.0 | 15.9 | 0.3 | 13.2 | 0.2 | 0.3 |
| Unknown_6 | 15.24 | 173.0839 | 948.2 | 23476.2 | 1121.0 | 1687.6 | 4707.8 | 61546.9 | 5829.7 | 4992.3 | 2814.2 | 437.5 | 660.3 | 1672.5 |
| Peak_4 | 16.1 | 214.9367 | 823.9 | 59526.2 | 690.5 | 100.1 | 29246.6 | 89131.8 | 1512.8 | 930.5 | 378.5 | 40.3 | 12.6 | 383.5 |
| Unknown_7 | 17.89 | 223.0653 | 17.5 | 15.6 | 0.3 | 36.9 | 55273.1 | 0.5 | 13.5 | 35.5 | 37.8 | 7.7 | 14.3 | 61.7 |
| Peak_5 | 19.14 | 198.941 | 7713.2 | 141863.3 | 21931.9 | 3955.6 | 87272.9 | 67474.2 | 1687.6 | 1245.2 | 1409.1 | 14.8 | 14.3 | 181.3 |

| | | | | | | | | | | | | | | |
|---|---|---|---|---|---|---|---|---|---|---|---|---|---|---|
| Unknown_8 | 22.99 | 294.8438 | 9040.0 | 51283.5 | 2031.5 | 216.6 | 108524.3 | 95611.1 | 22016.9 | 4651.5 | 5711.0 | 35.7 | 15.5 | 707.8 |
| Peak_6 | 23 | 292.8463 | 2509.6 | 25361.1 | 823.5 | 51.8 | 56786.0 | 49261.8 | 10854.5 | 1512.9 | 1994.3 | 0.5 | 9.1 | 194.9 |
| Unknown_9 | 23 | 296.8414 | 4506.8 | 24695.2 | 383.5 | 84.7 | 53160.4 | 45282.8 | 10245.8 | 1709.2 | 2036.7 | 19.8 | 10.2 | 247.6 |
| Unknown_10 | 26.29 | 448.0028 | 53.3 | 0.3 | 114.8 | 276.8 | 54787.3 | 168.9 | 44.4 | 52.3 | 82.0 | 51.6 | 35.7 | 54.1 |
| Peak_7 | 26.46 | 276.8508 | 88967.9 | 123965.6 | 24465.7 | 17498.9 | 159926.0 | 56668.6 | 37032.0 | 19082.7 | 27303.4 | 113.5 | 65.5 | 1610.3 |
| Unknown_11 | 26.47 | 280.8465 | 86405.4 | 122222.6 | 11525.3 | 17006.7 | 156080.5 | 51437.9 | 34432.9 | 19338.0 | 14321.2 | 78.2 | 68.7 | 1352.8 |
| Unknown_12 | 26.56 | 429.9919 | 56.0 | 88.9 | 88.1 | 6700.3 | 49511.2 | 0.3 | 46.8 | 26.8 | 0.1 | 25.5 | 12.3 | 22.4 |
| Peak_8 | 27.06 | 427.9931 | 53.6 | 35.6 | 88.2 | 7025.8 | 65253.0 | 32.0 | 14.3 | 17.2 | 7.8 | 0.5 | 7.4 | 22.8 |
| Unknown_13 | 27.06 | 429.9914 | 36.5 | 19.6 | 15.7 | 8860.7 | 91972.1 | 9.7 | 25.7 | 23.6 | 24.4 | 0.1 | 17.8 | 40.2 |
| Unknown_14 | 27.48 | 273.1246 | 29.4 | 144.2 | 55006.6 | 4152.8 | 120.1 | 108.4 | 56.5 | 91.3 | 23.9 | 28.1 | 19.4 | 107.6 |
| Unknown_15 | 27.89 | 413.9965 | 18.3 | 34.3 | 34.7 | 1577.7 | 94810.5 | 72.5 | 33.3 | 47.4 | 19.6 | 18.3 | 15.1 | 33.9 |
| Peak_9 | 28.28 | 413.9975 | 8.9 | 52.1 | 63.5 | 1427.1 | 60091.2 | 40.1 | 71.2 | 52.7 | 44.1 | 25.4 | 1.0 | 25.4 |
| Peak_10 | 28.98 | 354.9959 | 6342.3 | 156127.8 | 374241.3 | 176250.1 | 354.6 | 226369.3 | 12149.4 | 11010.7 | 10617.6 | 1043.1 | 859.0 | 13709.0 |
| Unknown_16 | 29.01 | 358.9915 | 1986.0 | 50746.1 | 122004.7 | 59291.4 | 167.7 | 76474.1 | 1394.1 | 1914.3 | 2898.2 | 259.1 | 266.5 | 2123.6 |
| Unknown_17 | 29.02 | 357.9962 | 716.1 | 28301.7 | 64471.6 | 34505.3 | 86.1 | 40753.6 | 968.2 | 1062.9 | 983.4 | 178.9 | 77.3 | 1335.5 |
| Unknown_1 | 29.1 | 336.984 | 7960.2 | 20330.4 | 55525.4 | 342.2 | 3786.7 | 45527.6 | 46389.2 | 10570.7 | 18438.1 | 77.0 | 258.3 | 10390.1 |

| | | | | | | | | | | | | | | |
|---|---|---|---|---|---|---|---|---|---|---|---|---|---|---|
| 8 | 1 | 9 | | | | | | | | | | | | |
| Unknown_19 | 29.11 | 338.9835 | 11029.4 | 32288.5 | 60375.2 | 938.5 | 5420.6 | 60765.7 | 59026.0 | 13479.0 | 23649.1 | 237.4 | 14.9 | 12702.3 |
| Unknown_20 | 29.39 | 322.9696 | 82705.5 | 26404.8 | 29588.3 | 17130.6 | 3425.3 | 64389.6 | 75366.5 | 15323.3 | 14070.1 | 270.7 | 890.2 | 23952.3 |
| Unknown_21 | 29.39 | 324.9671 | 106579.3 | 35044.0 | 35463.6 | 23727.3 | 10453.6 | 82964.4 | 95683.2 | 19573.2 | 18168.9 | 865.5 | 485.1 | 31087.7 |
| Unknown_22 | 29.62 | 209.0864 | 149.5 | 286.3 | 1149.8 | 1820.5 | 124768.0 | 293.3 | 158.6 | 123.0 | 134.5 | 285.9 | 161.7 | 131.9 |
| Unknown_23 | 29.76 | 358.9919 | 1918.4 | 13892.9 | 61147.1 | 125547.6 | 36.3 | 87086.9 | 11135.6 | 8773.3 | 8518.3 | 861.5 | 238.2 | 9579.0 |
| Unknown_24 | 29.77 | 354.9961 | 6800.1 | 42811.1 | 173442.8 | 362829.8 | 1392.8 | 282772.7 | 26344.4 | 28770.2 | 27145.7 | 4349.9 | 746.6 | 27953.3 |
| Unknown_25 | 29.79 | 357.9959 | 1123.8 | 7472.2 | 38868.8 | 65331.4 | 272.5 | 49489.5 | 2912.8 | 3677.5 | 4043.7 | 547.8 | 252.4 | 4517.1 |
| Unknown_26 | 29.8 | 275.07 | 350.1 | 5337.8 | 17086.7 | 46469.8 | 226.3 | 33284.9 | 2667.7 | 3441.4 | 2228.9 | 261.9 | 191.9 | 2954.0 |
| Peak_11 | 29.91 | 411.9811 | 44.3 | 0.2 | 0.4 | 1079.7 | 51057.1 | 0.5 | 33.5 | 15.8 | 81.6 | 0.5 | 23.8 | 51.2 |
| Unknown_27 | 29.93 | 340.9947 | 54038.6 | 24542.6 | 21376.7 | 4823.3 | 701.4 | 38871.9 | 57237.4 | 31203.3 | 101902.3 | 122.9 | 634.4 | 29812.1 |
| Unknown_28 | 29.94 | 336.9854 | 5323.7 | 38965.1 | 83830.5 | 720.8 | 1586.6 | 42802.2 | 40897.2 | 12457.3 | 11119.1 | 95.8 | 109.5 | 7825.2 |
| Unknown_29 | 29.96 | 452.0307 | 144.2 | 42.0 | 173.0 | 552.9 | 50903.9 | 122.5 | 117.2 | 34.3 | 64.3 | 0.1 | 0.7 | 0.7 |
| Peak_12 | 30.17 | 354.9953 | 591.9 | 4412.5 | 26461.0 | 112158.6 | 142.1 | 58245.1 | 1420.5 | 1991.2 | 2317.9 | 167.1 | 61.5 | 1507.4 |
| Unknown_30 | 30.35 | 452.03 | 80.7 | 18.3 | 72.0 | 306.2 | 43250.6 | 15.3 | 199.7 | 10.8 | 14.3 | 0.0 | 12.2 | 20.1 |
| Peak_13 | 30.69 | 252.964 | 123252.2 | 101876.4 | 135136.8 | 41317.4 | 4220.2 | 106476.7 | 180095.1 | 20791.9 | 20553.5 | 2863.7 | 240.2 | 12511.2 |

| | | | | | | | | | | | | | | |
|---|---|---|---|---|---|---|---|---|---|---|---|---|---|---|
| Unknown_31 | 30.69 | 256.9596 | 40538.5 | 33732.5 | 44949.5 | 14792.0 | 1310.0 | 36018.3 | 68367.4 | 6174.4 | 6478.2 | 934.1 | 286.9 | 3966.0 |
| Unknown_32 | 30.74 | 578.9744 | 74927.4 | 22133.8 | 40304.9 | 1972.1 | 134.4 | 3055.8 | 7641.5 | 174.2 | 170.6 | 42.2 | 64.7 | 311.1 |
| Peak_14 | 31.07 | 339.0001 | 76295.2 | 278336.4 | 191366.9 | 36995.3 | 5161.2 | 255107.9 | 541673.8 | 62692.4 | 70826.1 | 5286.8 | 1542.2 | 41967.2 |
| Peak_15 | 31.13 | 223.1011 | 545.4 | 5355.3 | 2762.1 | 2977.5 | 52442.6 | 7155.2 | 10640.8 | 855.4 | 724.7 | 253.3 | 42.7 | 509.5 |
| Unknown_33 | 31.14 | 385.061 | 219956.9 | 9495.0 | 2792.3 | 218.0 | 67.5 | 139.4 | 9327.6 | 22938.5 | 16359.3 | 70.7 | 105.2 | 1617.8 |
| Unknown_34 | 31.15 | 383.063 | 211529.5 | 10217.8 | 2939.1 | 106.0 | 152.1 | 338.3 | 1570.4 | 14697.2 | 8838.8 | 66.3 | 128.2 | 1278.4 |
| Unknown_35 | 31.45 | 364.0141 | 41.6 | 33.9 | 1377.5 | 3097.1 | 49601.7 | 131.3 | 193.6 | 131.1 | 50.6 | 49.0 | 0.5 | 235.0 |
| Unknown_36 | 31.57 | 352.9817 | 46538.7 | 28910.8 | 3172.9 | 704.2 | 1511.9 | 31693.4 | 57092.7 | 283.8 | 515.8 | 99.3 | 112.2 | 1538.1 |
| Unknown_37 | 31.57 | 354.9802 | 51167.2 | 39515.0 | 6688.9 | 1752.2 | 1470.9 | 48387.7 | 71074.7 | 868.4 | 903.9 | 90.2 | 52.1 | 1325.3 |
| Unknown_38 | 31.61 | 441.0685 | 26955.4 | 57537.3 | 9090.7 | 63.4 | 82.4 | 29922.1 | 73207.0 | 73851.8 | 40506.3 | 119.5 | 223.0 | 10577.6 |
| Unknown_39 | 31.61 | 443.0671 | 40060.5 | 74155.1 | 7251.5 | 115.8 | 150.2 | 39360.3 | 71924.5 | 92905.1 | 50606.9 | 299.1 | 28.5 | 14485.7 |
| Unknown_40 | 31.83 | 306.9747 | 121795.9 | 249233.3 | 172865.2 | 7905.3 | 279.8 | 240646.9 | 519842.0 | 33582.8 | 58295.5 | 445.0 | 1397.7 | 39996.9 |
| Unknown_41 | 31.83 | 310.9708 | 38978.8 | 81449.3 | 58346.9 | 2364.9 | 76.0 | 73382.7 | 184125.1 | 11813.4 | 22061.7 | 186.0 | 205.0 | 11986.3 |
| Unknown_42 | 31.89 | 336.9855 | 51551.6 | 23539.5 | 64430.4 | 32068.0 | 1220.9 | 13263.3 | 36553.6 | 15698.5 | 20617.0 | 425.7 | 158.6 | 17201.4 |
| Unknown_43 | 31.99 | 351.2176 | 101.5 | 406.3 | 76589.0 | 4686.1 | 62370.1 | 369.5 | 573.6 | 263.7 | 254.9 | 123.3 | 64.7 | 40.3 |
| Peak_16 | 32.2 | 320.990 | 673980. | 584988. | ######## | 111747.3 | 18967.5 | 558482. | ####### | 205766. | 323379. | 27124.5 | 22346.1 | 268381. |

| | | | | | | | | | | | | | | |
|---|---|---|---|---|---|---|---|---|---|---|---|---|---|---|
| | 8 | 3 | 9 | 2 | | | | 3 | # | 1 | 7 | | | 0 |
| Unknown_44 | 32.28 | 325.9895 | 36604.5 | 33291.4 | 68365.2 | 3331.2 | 437.3 | 26011.2 | 61511.0 | 11718.6 | 16104.4 | 716.2 | 548.2 | 13483.2 |
| Unknown_45 | 32.28 | 642.9908 | 58820.0 | 51410.8 | 82777.8 | 2697.0 | 52.2 | 28474.3 | 85125.5 | 8022.7 | 11296.2 | 55.1 | 34.7 | 13580.2 |
| Unknown_46 | 32.28 | 644.9872 | 146930.8 | 113237.6 | 221248.0 | 4986.6 | 235.2 | 88900.4 | 215391.1 | 16704.2 | 32808.7 | 143.5 | 64.6 | 32536.2 |
| Unknown_47 | 32.28 | 647.989 | 27973.0 | 25759.5 | 64782.7 | 1255.4 | 18.6 | 20074.0 | 62214.0 | 5260.3 | 8225.1 | 92.7 | 18.8 | 8634.6 |
| Unknown_48 | 32.28 | 648.9866 | 31384.6 | 47349.0 | 77851.5 | 2255.2 | 119.8 | 35654.7 | 90909.6 | 10630.9 | 16100.1 | 92.5 | 45.6 | 10558.3 |
| Unknown_49 | 32.53 | 308.8591 | 60241.4 | 60037.7 | 334.2 | 64.9 | 291.9 | 3577.1 | 20916.9 | 1436.3 | 5270.6 | 8.3 | 14.5 | 270.6 |
| Unknown_50 | 32.63 | 380.9952 | 8290.7 | 34831.4 | 72109.1 | 1390.2 | 30.7 | 33649.1 | 70327.5 | 23033.4 | 20769.4 | 151.2 | 114.9 | 10982.9 |
| Unknown_51 | 32.64 | 378.9975 | 8214.7 | 26902.8 | 54402.3 | 1718.3 | 92.2 | 27826.2 | 57277.1 | 19218.4 | 16046.1 | 111.6 | 108.3 | 8774.2 |
| Unknown_52 | 32.81 | 597.1974 | 150.7 | 105.7 | 46144.2 | 93.2 | 30.8 | 11.1 | 17.5 | 27.9 | 23.9 | 39.6 | 28.6 | 33.6 |
| Unknown_53 | 32.84 | 383.0619 | 157408.5 | 6921.8 | 5569.8 | 229.6 | 66.6 | 163.0 | 1131.5 | 17651.0 | 2879.3 | 117.7 | 0.1 | 493.2 |
| Unknown_54 | 32.85 | 385.0604 | 196577.1 | 12553.7 | 11141.9 | 141.9 | 59.3 | 368.1 | 6241.6 | 21225.7 | 6717.3 | 102.4 | 142.2 | 637.9 |
| Unknown_55 | 33.15 | 320.99 | 48099.7 | 28839.3 | 50326.8 | 5227.2 | 2380.0 | 28615.9 | 56186.0 | 13144.8 | 18079.8 | 380.8 | 206.3 | 14681.0 |
| Unknown_56 | 33.15 | 322.9882 | 65745.8 | 39604.1 | 69256.0 | 7276.5 | 3614.7 | 38098.3 | 77508.5 | 19863.3 | 22156.3 | 667.2 | 606.7 | 20946.0 |
| Unknown_57 | 33.32 | 336.9852 | 88547.3 | 25998.1 | 5990.9 | 1897.5 | 3606.7 | 25434.6 | 56647.9 | 19371.0 | 25447.5 | 272.1 | 244.6 | 20002.4 |
| Unknown_58 | 33.32 | 338.9835 | 116266.4 | 37248.7 | 6243.0 | 3107.9 | 4534.1 | 32758.2 | 72172.2 | 26057.7 | 31112.7 | 252.7 | 197.7 | 25925.7 |

| | | | | | | | | | | | | | | |
|---|---|---|---|---|---|---|---|---|---|---|---|---|---|---|
| Unknown_59 | 33.46 | 322.9885 | 46893.7 | 14768.3 | 46476.0 | 7694.4 | 25851.2 | 16572.5 | 52303.8 | 18742.9 | 9607.4 | 443.2 | 739.0 | 6200.0 |
| Unknown_60 | 33.61 | 311.1858 | 644.8 | 6557.1 | 46566.0 | 1794.8 | 5638.1 | 5704.2 | 16161.1 | 1377.4 | 5268.6 | 265.9 | 150.1 | 3175.1 |
| Unknown_61 | 34.07 | 385.205 | 76.5 | 114.3 | 743.3 | 2791.1 | 45282.7 | 247.5 | 171.7 | 92.5 | 161.6 | 17.3 | 70.2 | 146.7 |
| Unknown_62 | 34.16 | 457.0827 | 16704.1 | 41122.3 | 342.8 | 60.7 | 0.8 | 683.3 | 51213.8 | 40572.8 | 6462.8 | 82.0 | 0.6 | 1234.2 |
| Unknown_63 | 34.17 | 455.0823 | 12453.9 | 31561.2 | 482.3 | 109.5 | 48.1 | 1001.9 | 50893.8 | 30771.0 | 5580.4 | 81.8 | 98.7 | 665.3 |
| Unknown_64 | 34.2 | 239.1291 | 8270.5 | 9962.4 | 52190.9 | 2162.6 | 875.4 | 1208.0 | 4942.0 | 3931.7 | 2823.5 | 1748.0 | 1476.0 | 2066.3 |
| Peak_17 | 34.21 | 385.0422 | 7303.8 | 36527.4 | 23557.3 | 4029.1 | 85.4 | 1707.3 | 55179.6 | 2231.0 | 2118.8 | 60.6 | 26.4 | 327.3 |
| Unknown_65 | 34.22 | 387.0405 | 1178.1 | 42519.2 | 29342.5 | 5092.9 | 184.1 | 1621.3 | 57768.7 | 1305.2 | 337.9 | 59.7 | 41.0 | 105.7 |
| Unknown_66 | 34.27 | 399.0751 | 41824.6 | 3860.1 | 15446.2 | 114.6 | 74.5 | 173.3 | 10853.0 | 3899.4 | 121.3 | 84.5 | 60.0 | 1250.9 |
| Unknown_67 | 34.36 | 469.0654 | 19811.2 | 31404.3 | 1631.9 | 34.7 | 38.4 | 6955.8 | 63182.0 | 52553.0 | 7858.9 | 48.7 | 88.0 | 2189.0 |
| Unknown_68 | 34.36 | 471.0625 | 23552.2 | 43226.0 | 1742.4 | 49.7 | 11.9 | 8883.8 | 78554.3 | 68323.3 | 8989.2 | 101.3 | 46.8 | 3226.5 |
| Unknown_69 | 34.38 | 369.0105 | 112208.1 | 15600.0 | 58.8 | 21.1 | 32.4 | 10078.3 | 4096.5 | 3723.6 | 0.0 | 0.7 | 66.1 | 146.3 |
| Unknown_70 | 34.39 | 333.2074 | 53.6 | 158.6 | 66161.5 | 7563.5 | 11427.9 | 0.9 | 0.7 | 50.6 | 79.9 | 0.4 | 0.1 | 70.6 |
| Unknown_71 | 34.44 | 287.2235 | 1872.5 | 11557.9 | 11607.7 | 243.9 | 1976.2 | 5561.4 | 124409.7 | 13643.4 | 14362.3 | 687.1 | 212.1 | 28762.5 |
| Peak_18 | 34.53 | 460.9735 | 26861.8 | 47220.4 | 2555.3 | 25.0 | 38.9 | 7802.0 | 34078.6 | 25933.2 | 13796.2 | 54.0 | 6.9 | 676.7 |
| Unknown_7 | 34.9 | 482.955 | 23731.0 | 22863.5 | 86.1 | 221.6 | 182.4 | 3639.7 | 21779.1 | 19221.3 | 41270.1 | 587.2 | 191.2 | 3160.4 |

| | | | | | | | | | | | | | | |
|---|---|---|---|---|---|---|---|---|---|---|---|---|---|---|
| 2 | 6 | 2 | | | | | | | | | | | | |
| Peak_19 | 34.99 | 444.9794 | 82580.5 | 99711.7 | 362.9 | 274.8 | 331.0 | 241.8 | 10993.4 | 52923.7 | 296905.4 | 739.7 | 529.6 | 9821.0 |
| Unknown_73 | 34.99 | 449.9782 | 21479.5 | 27127.7 | 15.1 | 51.0 | 18.5 | 0.7 | 3554.6 | 14101.6 | 78657.4 | 136.3 | 107.1 | 2210.3 |
| Unknown_74 | 34.99 | 492.9833 | 27255.8 | 37659.0 | 125.1 | 541.2 | 456.8 | 225.2 | 1315.8 | 19313.3 | 126873.8 | 1004.9 | 548.9 | 4159.2 |
| Unknown_75 | 35 | 484.9534 | 19312.1 | 21367.6 | 164.2 | 104.8 | 260.4 | 5572.3 | 22654.3 | 23083.8 | 40008.3 | 578.8 | 280.8 | 5262.1 |
| Unknown_76 | 35.05 | 413.0733 | 5248.5 | 137496.4 | 1351.2 | 60.7 | 16.5 | 1462.8 | 30385.9 | 2466.0 | 3525.6 | 159.2 | 107.8 | 11532.8 |
| Unknown_77 | 35.05 | 415.0718 | 11531.0 | 136870.8 | 1558.6 | 18.5 | 14.5 | 1664.2 | 36833.3 | 5238.9 | 7782.7 | 121.0 | 24.4 | 26303.4 |
| Unknown_78 | 35.06 | 417.0684 | 1525.0 | 50882.2 | 505.8 | 20.9 | 48.1 | 846.4 | 10190.9 | 645.2 | 3135.5 | 106.4 | 60.2 | 5861.6 |
| Unknown_79 | 35.25 | 328.7631 | 110346.9 | 1227.9 | 1919.8 | 218.1 | 30.4 | 70.8 | 51.9 | 82.5 | 91.7 | 14.7 | 41.3 | 77.4 |
| Unknown_80 | 35.25 | 330.7608 | 104806.9 | 2091.3 | 1624.2 | 106.6 | 41.3 | 47.7 | 13.0 | 68.4 | 144.5 | 15.4 | 26.0 | 29.6 |
| Unknown_81 | 35.33 | 476.952 | 41484.2 | 63413.7 | 0.4 | 0.3 | 45.8 | 284.9 | 7643.4 | 4379.8 | 670.4 | 47.1 | 100.5 | 342.4 |
| Unknown_82 | 35.37 | 320.9898 | 43575.2 | 13122.0 | 9152.9 | 919.4 | 553.0 | 4917.3 | 45514.8 | 30512.7 | 3632.0 | 362.5 | 259.9 | 3094.7 |
| Unknown_83 | 35.37 | 322.9878 | 65282.8 | 18880.3 | 13307.2 | 1477.5 | 828.3 | 5782.4 | 59138.7 | 37353.6 | 4252.3 | 509.9 | 565.3 | 2726.6 |
| Peak_20 | 35.56 | 287.2223 | 899.4 | 549.5 | 9160.3 | 67985.9 | 68081.8 | 765.4 | 2823.1 | 1670.9 | 1538.1 | 605.5 | 920.4 | 1432.2 |
| Unknown_84 | 35.69 | 365.0525 | 51348.3 | 17263.8 | 36818.6 | 280.1 | 44.3 | 151.5 | 1042.0 | 4803.3 | 18487.5 | 56.4 | 64.7 | 895.3 |
| Unknown_85 | 35.7 | 267.1969 | 354.0 | 7939.5 | 178830.9 | 2945.6 | 18089.9 | 300.0 | 6919.3 | 2916.6 | 70.0 | 46.1 | 25.7 | 1318.1 |

| | | | | | | | | | | | | | | |
|---|---|---|---|---|---|---|---|---|---|---|---|---|---|---|
| Unknown_86 | 35.7 | 367.05 | 128738.6 | 30283.6 | 69309.9 | 899.5 | 197.8 | 256.9 | 6884.5 | 25137.4 | 53349.3 | 399.7 | 316.4 | 3995.7 |
| Unknown_87 | 35.85 | 333.2072 | 177.3 | 495.3 | 116791.7 | 2078.3 | 1135.6 | 75.1 | 179.1 | 375.2 | 132.4 | 14.4 | 55.6 | 95.0 |
| Peak_21 | 36.28 | 527.2548 | 1180.1 | 22542.4 | 7247.3 | ######## | 40247.1 | 1249.0 | 1672.8 | 39761.1 | 3643.4 | 1637.1 | 357.5 | 3366.0 |
| Peak_22 | 36.73 | 269.213 | 15776.1 | 71958.3 | 203388.8 | 14467.5 | 10837.3 | 574.7 | 38965.8 | 189398.2 | 6526.8 | 8721.4 | 1135.8 | 163355.2 |
| Unknown_88 | 36.74 | 270.2157 | 2474.2 | 14127.7 | 36732.1 | 1408.3 | 1535.5 | 81.9 | 5508.0 | 45199.3 | 981.4 | 942.5 | 25.4 | 22773.2 |
| Unknown_89 | 36.79 | 460.9575 | 15496.2 | 81506.2 | 1178.0 | 50.6 | 0.0 | 17.0 | 6222.6 | 81929.9 | 2144.4 | 21.8 | 15.7 | 670.5 |
| Unknown_90 | 36.97 | 297.1539 | 43101.8 | 47069.5 | 22778.5 | 71752.3 | 53971.4 | 37657.5 | 38559.0 | 185227.9 | 38936.0 | 46084.2 | 40451.4 | 43486.2 |
| Unknown_91 | 37.24 | 267.197 | 82100.9 | 477603.8 | 103994.7 | 1677.1 | 762.0 | 729.4 | 43233.9 | 220644.2 | 24223.0 | 7385.0 | 839.9 | 146960.3 |
| Peak_23 | 37.62 | 488.9695 | 243917.1 | 498408.9 | 34552.5 | 948.8 | 246.7 | 1456.2 | 4861.8 | 502957.2 | 79975.7 | 2017.6 | 1252.1 | 11732.4 |
| Peak_24 | 37.75 | 492.9825 | 491462.7 | 957479.6 | 269255.4 | 12520.5 | 1738.8 | 2768.8 | 17937.7 | ######## | 642621.5 | 40845.2 | 23182.4 | 48911.5 |
| Unknown_92 | 37.79 | 444.9782 | 27970.9 | 283708.1 | 95207.1 | 6321.7 | 1065.5 | 1183.5 | 6207.2 | 419238.3 | 260777.7 | 15204.3 | 7220.4 | 15007.0 |
| Unknown_93 | 37.79 | 446.9772 | 58851.4 | 721335.9 | 234472.2 | 13154.8 | 1516.3 | 2242.8 | 14350.9 | 733030.8 | 471702.4 | 35128.4 | 15791.5 | 32120.2 |
| Unknown_94 | 37.81 | 448.975 | 39117.2 | 431665.3 | 164366.0 | 8463.7 | 1333.4 | 1349.1 | 9277.2 | 521688.7 | 341089.1 | 23451.6 | 13020.3 | 23152.3 |
| Unknown_95 | 37.87 | 447.9806 | 6439.8 | 96660.9 | 36835.5 | 1892.8 | 255.9 | 472.8 | 1539.4 | 134564.3 | 87997.5 | 4442.5 | 1766.1 | 3407.3 |
| Peak_26 | 37.88 | 533.9926 | 6178.1 | 29984.9 | 17567.1 | 1776.6 | 211.3 | 163.8 | 1539.8 | 44223.2 | 32825.8 | 3988.5 | 1410.6 | 2391.4 |
| Unknown_9 | 38.1 | 309.174 | 75928.9 | 47767.5 | 71132.3 | 100870.1 | 43240.6 | 35223.0 | 34649.5 | 49383.0 | 49067.8 | 34196.2 | 36631.5 | 42873.7 |

| | | | | | | | | | | | | | | |
|---|---|---|---|---|---|---|---|---|---|---|---|---|---|---|
| 6 | | 7 | | | | | | | | | | | | |
| Unknown_9 | 38.4 | | | | | | | | | | | | | |
| 7 | 3 | 643.984 | 262.8 | 38.3 | 46418.8 | 0.8 | 0.6 | 67.4 | 412.0 | 4198.4 | 7727.3 | 83.8 | 81.7 | 413.3 |
| Unknown_9 | 38.4 | 641.986 | | | | | | | | | | | | |
| 8 | 4 | 4 | 190.3 | 33.8 | 48704.6 | 111.5 | 65.3 | 48.2 | 203.9 | 5998.0 | 7671.0 | 61.2 | 95.8 | 234.8 |
| Unknown_9 | 38.5 | | | | | | | | | | | | | |
| 9 | 8 | 669.317 | 269.6 | 5038.5 | 117.2 | 62691.1 | 14153.5 | 227.7 | 308.6 | 4828.0 | 162.5 | 114.8 | 79.6 | 824.2 |
| Unknown_1 | 38.6 | 623.272 | | | | | | | | | | | | |
| 00 | 1 | 7 | 592.5 | 483.3 | 167.4 | 69213.4 | 22982.2 | 253.2 | 295.2 | 1224.9 | 313.4 | 331.2 | 367.5 | 359.4 |
| Unknown_1 | 38.6 | 558.285 | | | | | | | | | | | | |
| 01 | 3 | 9 | 260.5 | 41.6 | 426.8 | 195714.3 | 16928.5 | 92.5 | 167.4 | 809.5 | 177.6 | 119.5 | 12.8 | 251.1 |
| | | 555.284 | | | | | 669132. | | | | | | | |
| Peak_25 | 38.7 | 4 | 4069.0 | 68.3 | 35880.1 | ######## | 3 | 2821.0 | 1861.0 | 32928.9 | 2124.0 | 1274.2 | 1213.5 | 7231.2 |
| | | 537.330 | | | | | | | | | | | | |
| Peak_27 | 38.8 | 4 | 0.1 | 0.1 | 16380.0 | 6937.0 | 0.1 | 0.1 | 0.1 | 0.1 | 0.1 | 0.1 | 0.1 | 0.1 |
| | | 537.332 | | | | | | | | | | | | |
| Peak_28 | 39.2 | 3 | 0.1 | 0.1 | 40222.0 | 42561.0 | 0.1 | 0.1 | 0.1 | 0.1 | 0.1 | 0.1 | 0.1 | 0.1 |
| Unknown_1 | 39.3 | 397.227 | | | | | | | | | | | | |
| 02 | 3 | 2 | 19353.8 | 43937.7 | 14225.7 | 25031.6 | 14140.4 | 22184.1 | 18482.0 | 15436.1 | 13950.4 | 20076.0 | 16306.7 | 19576.8 |
| Unknown_1 | 39.6 | 483.273 | | | | | | | | | | | | |
| 03 | 4 | 5 | 1456.6 | 1246.7 | 1059.9 | 534574.0 | 49020.2 | 401.1 | 175.3 | 557.7 | 2003.6 | 254.0 | 178.8 | 550.0 |
| Unknown_1 | 39.6 | 484.276 | | | | | | | | | | | | |
| 04 | 4 | 7 | 106.1 | 482.9 | 444.8 | 135454.0 | 15128.8 | 105.8 | 76.8 | 178.1 | 434.9 | 64.7 | 55.2 | 75.0 |
| Unknown_1 | 39.7 | 311.199 | 122587. | 244024. | | | | | | | 149625. | | | |
| 05 | 3 | 6 | 3 | 5 | 61912.3 | 37291.8 | 24622.2 | 51221.5 | 53311.3 | 65579.4 | 2 | 86956.0 | 78902.5 | 90879.8 |
| Unknown_1 | 39.7 | 312.203 | | | | | | | | | | | | |
| 06 | 3 | 9 | 25234.6 | 53150.9 | 14296.4 | 9415.7 | 8864.3 | 9451.3 | 9772.6 | 12081.2 | 32972.3 | 16216.1 | 15571.3 | 18569.0 |
| Unknown_1 | 40.1 | 446.312 | | | | | | | | | | | | |
| 07 | 5 | 4 | 1832.3 | 11359.6 | 63241.3 | 1071.5 | 171.5 | 91.3 | 107.0 | 340.4 | 18813.5 | 317.0 | 251.1 | 679.5 |
| | 40.3 | 474.990 | 128654. | 351979. | | | | | | | 662968. | | | 552815. |
| Peak_29 | 2 | 2 | 6 | 7 | 21282.3 | 3042.4 | 1221.5 | 1719.5 | 769.6 | 1336.0 | 6 | 18510.0 | 4917.4 | 8 |

| | | | | | | | | | | | | | | |
|---|---|---|---|---|---|---|---|---|---|---|---|---|---|---|
| Unknown_108 | 40.32 | 478.9859 | 211007.0 | 756439.6 | 34722.4 | 5824.1 | 1371.3 | 2961.5 | 916.0 | 1320.9 | ######## | 29989.3 | 9104.4 | 974328.2 |
| Peak_30 | 40.48 | 271.2283 | 14850.5 | 32426.3 | 36270.4 | 21146.0 | 3064.5 | 1136.9 | 779.6 | 1492.9 | 52841.8 | 3103.7 | 2100.7 | 28561.3 |
| Unknown_109 | 41.16 | 271.2283 | 19396.4 | 24056.3 | 122888.1 | 525899.9 | 1060.5 | 4762.4 | 3916.1 | 4392.2 | 10037.0 | 5025.4 | 4682.5 | 28727.6 |
| Unknown_110 | 41.16 | 272.232 | 3503.0 | 4202.4 | 21834.7 | 97323.8 | 445.2 | 535.0 | 476.9 | 433.6 | 871.6 | 545.1 | 788.1 | 6193.7 |
| Peak_31 | 41.5 | 340.2861 | 27525.44 | 20996.75 | 50779.69 | 60374.94 | 45277.18 | 32678.65 | 29432.79 | 28378.37 | 48020.78 | 34385.97 | 27148.9 | 78159.01 |
| Peak_32 | 42 | 384.3129 | 0.1 | 65.0 | 2173.0 | 0.1 | 0.1 | 0.1 | 0.1 | 0.1 | 0.1 | 0.1 | 0.1 | 586.0 |
| Peak_33 | 42.3 | 384.3135 | 0.1 | 75.0 | 1358.0 | 0.1 | 0.1 | 0.1 | 0.1 | 0.1 | 0.1 | 0.1 | 0.1 | 137.0 |
| Unknown_111 | 43.45 | 473.2845 | 3535.6 | 936.2 | 14920.9 | 18171.8 | 704.0 | 11188.6 | 10146.1 | 13977.2 | 13856.4 | 25105.3 | 4099.4 | 40738.1 |
| Unknown_112 | 43.76 | 255.234 | 96845.1 | 241164.1 | 58041.0 | 35387.3 | 2037.3 | 22069.3 | 22436.4 | 22974.2 | 22707.3 | 27709.6 | 28040.3 | 282803.6 |
| Peak_34 | 44.1 | 309.2428 | 194.3 | 238.3 | 154145.3 | 43140.1 | 1650.9 | 13.9 | 201.0 | 0.1 | 32.6 | 55.0 | 38.7 | 45.5 |
| Unknown_113 | 44.27 | 281.2491 | 42806.8 | 94310.8 | 26112.9 | 22587.6 | 7796.7 | 24221.7 | 20649.2 | 16455.2 | 15079.3 | 20951.7 | 22726.3 | 16414.4 |
| Peak_35 | 44.5 | 765.4752 | 0.1 | 0.1 | 0.1 | 2911.0 | 14798.0 | 7524.0 | 9263.0 | 6121.0 | 137.0 | 61.0 | 0.1 | 0.1 |
| Activity Score | - | - | 115 | 120 | 10 | 20 | 16 | 0.1 | 0.1 | 0.1 | 130 | 120 | 16 | 16 |

**S6:** List of entries used in the statistical correlation analyses between compounds detected in the HPLC-MS chromatograms (negative ion mode) and the schistosomicidal activity score.

| Entry | Rt | *m/z* | Ld-Hex | Ld-DCM | Ld-AcOEt | Ld-MeOH | Ld-$H_2O$ | Ld-F1 | Ld-F2 | Ld-F3 | Ld-F4 | Ld-F5 | Ld-F6 | Ld-F7 |
|---|---|---|---|---|---|---|---|---|---|---|---|---|---|---|
| | | | Active | Active | Inactive | Inactive | Inactive | Inactive | Inactive | Active | Active | Inactive | Active | Inactive |
| Unknown 1 | 1.34 | 253.0924 | 15.0 | 100.0 | 3495.5 | 32079.2 | 105862.2 | 410.1 | 94.9 | 23.6 | 43.0 | 0.1 | 0.1 | 39.2 |
| Unknown 2 | 1.34 | 289.0694 | 0.1 | 60.4 | 136.2 | 4882.2 | 125409.4 | 137.2 | 0.1 | 0.1 | 0.1 | 10.3 | 15.6 | 0.1 |
| Unknown 3 | 1.34 | 152.0397 | 0.1 | 105.5 | 1326.3 | 1845.3 | 222059.9 | 157.7 | 41.8 | 37.0 | 0.1 | 0.1 | 17.3 | 52.0 |
| Unknown 4 | 1.36 | 168.0342 | 54.6 | 21.0 | 84.8 | 144.5 | 50815.6 | 60.3 | 27.0 | 44.9 | 0.1 | 0.1 | 14.2 | 13.1 |
| Unknown 5 | 2.28 | 172.8432 | 105.2 | 51.1 | 86.4 | 128.0 | 60729.8 | 60.7 | 14.1 | 79.9 | 12.0 | 0.1 | 0.1 | 16.5 |
| Unknown 6 | 3.76 | 241.0844 | 0.1 | 0.1 | 70.2 | 60.0 | 63141.4 | 235.6 | 0.1 | 25.7 | 0.1 | 9.5 | 30.4 | 0.1 |
| Unknown 7 | 3.77 | 188.0932 | 46.5 | 0.1 | 0.1 | 21.2 | 70155.6 | 0.1 | 0.1 | 0.1 | 0.1 | 0.1 | 0.1 | 0.1 |
| Peak 1 | 4.17 | 303.1925 | 0.1 | 0.1 | 0.1 | 17.5 | 299178.0 | 10.8 | 0.1 | 0.1 | 0.1 | 0.1 | 0.1 | 0.1 |
| Unknown 8 | 4.17 | 244.1189 | 0.1 | 0.1 | 37.5 | 80.6 | 77573.9 | 17.4 | 0.1 | 19.3 | 0.1 | 0.1 | 0.1 | 0.1 |
| Unknown 9 | 12.83 | 241.1189 | 165.5 | 86.2 | 130.8 | 55.4 | 59640.6 | 75.6 | 70.7 | 86.5 | 22.8 | 18.0 | 50.4 | 15.4 |
| Peak 2 | 18.38 | 283.1548 | 1294.2 | 46640.8 | 96588.4 | 25141.0 | 1715.7 | 126.6 | 116.8 | 130.8 | 9.7 | 91.4 | 14.7 | 40.0 |
| Peak 3 | 21.77 | 283.1549 | 2277.6 | 26948.1 | 24894.0 | 365.5 | 45875.6 | 80030.8 | 34987.8 | 2381.8 | 560.1 | 479.2 | 469.1 | 83.6 |
| Peak 4 | 26.41 | 209.0853 | 85.5 | 158.8 | 176.0 | 486.2 | 425668.2 | 300.1 | 95.1 | 37.8 | 31.8 | 104.5 | 104.5 | 44.0 |
| Unknown 10 | 26.48 | 278.8478 | 42950.0 | 4296.0 | 310.6 | 646.4 | 70411.0 | 6393.7 | 167.5 | 287.1 | 119.4 | 25.9 | 0.1 | 34.0 |
| Peak 5 | 29.67 | 215.1282 | 4600.4 | 15093.5 | 10099.7 | 7314.3 | 7979.1 | 32844.4 | 9915.6 | 12797.3 | 42604.0 | 7581.2 | 9474.1 | 2981.1 |

| | | | | | | | | | | | | | | |
|---|---|---|---|---|---|---|---|---|---|---|---|---|---|---|
| Peak 6 | 31.11 | 223.1010 | 214.8 | 354.1 | 72.0 | 1372.2 | 119058.8 | 635.9 | 102.9 | 178.1 | 77.0 | 61.2 | 60.1 | 66.7 |
| Unknown 11 | 31.43 | 180.1160 | 1334.2 | 12417.4 | 138745.9 | 487.5 | 13502.9 | 13834.4 | 8833.4 | 641.4 | 70.9 | 141.5 | 96.5 | 59.5 |
| Peak 7 | 31.43 | 267.1600 | 33396.3 | 116550.9 | 1127636.6 | 7243.9 | 91247.9 | 172684.9 | 78821.1 | 9042.1 | 3692.2 | 1897.8 | 959.7 | 267.0 |
| Peak 8 | 31.63 | 393.0106 | 59460.6 | 103615.6 | 5023.0 | 1064.3 | 578.0 | 82393.9 | 45380.6 | 40608.8 | 8408.9 | 10912.3 | 1642.7 | 324.3 |
| Unknown 12 | 32.02 | 351.2172 | 757.5 | 5614.6 | 269883.5 | 28029.0 | 73159.2 | 2249.9 | 205.5 | 216.2 | 364.0 | 230.4 | 17.2 | 81.0 |
| Peak 9 | 32.26 | 267.1594 | 41511.0 | 49366.6 | 989730.1 | 5305.8 | 85438.9 | 47260.5 | 84620.0 | 3701.0 | 1848.2 | 1900.8 | 575.9 | 255.7 |
| Peak 10 | 32.66 | 379.0311 | 53601.6 | 117262.6 | 315.9 | 176.5 | 98.6 | 154942.6 | 102483.7 | 56336.8 | 17079.9 | 4041.9 | 1081.3 | 193.8 |
| Unknown 13 | 33.00 | 267.1602 | 28474.0 | 12025.8 | 101167.2 | 5423.2 | 62362.1 | 4802.4 | 22207.5 | 7860.8 | 738.6 | 1048.1 | 810.7 | 486.1 |
| Unknown 14 | 33.28 | 265.1441 | 73895.6 | 2060.9 | 1258.3 | 674.5 | 1830.6 | 1102.1 | 1458.9 | 1610.5 | 950.6 | 288.7 | 1313.9 | 3709.2 |
| Unknown 15 | 33.62 | 311.1869 | 1507.1 | 15264.5 | 118473.5 | 6093.9 | 16467.4 | 15005.4 | 19368.2 | 1299.5 | 4330.8 | 3119.7 | 763.3 | 198.0 |
| Peak 11 | 33.76 | 349.0211 | 31095.2 | 42611.0 | 6149.9 | 821.6 | 60.7 | 9174.9 | 26053.6 | 13247.2 | 4509.9 | 138.0 | 85.1 | 14.1 |
| Unknown 16 | 33.93 | 365.2323 | 89.1 | 0.1 | 192941.5 | 17934.0 | 26251.1 | 0.1 | 45.2 | 12.1 | 46.1 | 40.2 | 48.1 | 37.2 |
| Unknown 17 | 34.44 | 409.0402 | 49743.7 | 9009.7 | 0.1 | 0.1 | 15.5 | 240.7 | 298.9 | 499.4 | 137.4 | 0.1 | 16.0 | 50.6 |
| Peak 12 | 34.85 | 347.0049 | 23411.7 | 40799.2 | 78.6 | 36.7 | 10.0 | 15187.1 | 23435.1 | 27321.0 | 1202.9 | 63.1 | 13.5 | 32.2 |
| Unknown 18 | 36.05 | 699.3596 | 285.8 | 354.7 | 625.0 | 51859.9 | 58.4 | 193.2 | 244.1 | 213.9 | 122.6 | 71.9 | 35.7 | 40.7 |
| Unknown 19 | 36.05 | 746.3678 | 21.6 | 62.6 | 927.6 | 89065.5 | 82.8 | 170.7 | 9.6 | 14.9 | 45.1 | 34.0 | 30.0 | 0.1 |
| Peak 13 | 36.28 | 527.2537 | 615.6 | 11332.3 | 10095.2 | 1315036.3 | 62243.5 | 1512.8 | 853.1 | 7295.8 | 445.6 | 572.0 | 1985.6 | 1216.7 |
| Unknown 20 | 36.29 | 697.3645 | 444.1 | 436.5 | 404.3 | 61791.5 | 498.3 | 124.8 | 173.0 | 115.3 | 115.0 | 86.1 | 55.3 | 0.1 |

| | | | | | | | | | | | | | | |
|---|---|---|---|---|---|---|---|---|---|---|---|---|---|---|
| Unknown 21 | 36.45 | 586.3147 | 680.3 | 1277.9 | 2147.5 | 240446.5 | 1136.7 | 912.7 | 497.3 | 704.6 | 284.9 | 511.4 | 434.5 | 189.3 |
| Peak 14 | 36.78 | 601.2686 | 250.7 | 509.2 | 2597.0 | 987488.5 | 8481.2 | 788.1 | 243.2 | 200.3 | 200.6 | 377.3 | 139.3 | 91.4 |
| Unknown 22 | 36.85 | 315.2528 | 5850.2 | 47597.4 | 1185.3 | 33590.0 | 7630.0 | 3210.9 | 6892.9 | 65046.7 | 11865.2 | 32933.4 | 12629.1 | 6027.3 |
| Peak 15 | 37.04 | 287.2229 | 211.5 | 726.5 | 57838.0 | 118944.4 | 199415.2 | 161.5 | 231.4 | 496.8 | 531.6 | 940.1 | 412.6 | 260.3 |
| Peak 16 | 37.08 | 285.2071 | 53916.2 | 325284.1 | 22844.6 | 749.2 | 136.9 | 83.6 | 1570.9 | 267230.6 | 3902.0 | 247000.4 | 6688.1 | 2750.2 |
| Unknown 23 | 37.15 | 501.3904 | 0.1 | 125.2 | 103.4 | 141114.9 | 9668.5 | 1316.5 | 196.8 | 268.5 | 456.5 | 0.1 | 69.3 | 14.3 |
| Peak 17 | 37.36 | 317.2125 | 1370.3 | 1877.3 | 3180603.8 | 158775.4 | 33566.8 | 480.1 | 330.3 | 1218.5 | 363.2 | 633.9 | 254.4 | 174.7 |
| Unknown 24 | 37.50 | 588.3302 | 770.1 | 488.9 | 909.3 | 266914.7 | 2008.3 | 525.1 | 248.4 | 565.4 | 262.0 | 367.9 | 327.9 | 165.4 |
| Peak 18 | 37.63 | 653.3758 | 291.2 | 744.8 | 4414.6 | 293466.2 | 25787.6 | 426.5 | 333.1 | 2665.5 | 437.7 | 310.7 | 161.8 | 107.3 |
| Unknown 25 | 37.64 | 481.2580 | 414.9 | 1318.0 | 1481.9 | 65164.6 | 845.5 | 670.2 | 286.6 | 566.4 | 550.0 | 233.3 | 380.9 | 66.2 |
| Unknown 26 | 37.65 | 654.3790 | 366.5 | 410.1 | 1971.5 | 109251.1 | 7183.3 | 496.7 | 103.6 | 1172.8 | 251.4 | 437.1 | 90.7 | 115.8 |
| Unknown 27 | 37.65 | 701.3870 | 514.2 | 647.9 | 2993.8 | 119723.2 | 10380.6 | 462.0 | 221.1 | 799.1 | 435.1 | 232.8 | 152.0 | 214.7 |
| Unknown 28 | 37.96 | 603.2848 | 1047.7 | 212.7 | 0.1 | 105904.1 | 905.5 | 417.7 | 205.0 | 518.0 | 342.5 | 42.3 | 86.0 | 150.5 |
| Unknown 29 | 38.13 | 726.3976 | 70.3 | 197.8 | 413.1 | 74652.6 | 379.8 | 185.5 | 46.9 | 64.5 | 39.7 | 91.4 | 72.7 | 22.2 |
| Peak 19 | 38.54 | 319.2293 | 611.3 | 865.5 | 610811.9 | 13677.4 | 3267.9 | 131.1 | 61.9 | 268.5 | 458.8 | 375.7 | 395.7 | 121.4 |
| Peak 20 | 38.54 | 555.2853 | 2057.3 | 33838.4 | 53298.6 | 11250180.7 | 136014.8 | 8178.0 | 3828.9 | 18058.0 | 14966.2 | 11316.6 | 10794.0 | 5695.5 |
| Peak 21 | 38.55 | 319.2281 | 1381.7 | 7338.6 | 2684279.0 | 25974.6 | 20634.6 | 247.7 | 379.8 | 651.1 | 2036.1 | 1110.0 | 3583.1 | 364.6 |
| Unknown 30 | 38.58 | 558.2878 | 365.6 | 1108.9 | 118.5 | 253692.2 | 25854.9 | 195.3 | 124.7 | 231.5 | 215.0 | 187.8 | 173.4 | 105.6 |

| | | | | | | | | | | | | | | | |
|---|---|---|---|---|---|---|---|---|---|---|---|---|---|---|---|
| Unknown 31 | 38.63 | 623.2726 | 1087.0 | 479.3 | 548.4 | 97480.3 | 34014.5 | 613.6 | 316.9 | 626.7 | 443.1 | 318.8 | 446.1 | 284.7 |
| Unknown 32 | 38.75 | 481.2580 | 950.8 | 1838.3 | 1856.1 | 229923.6 | 36839.6 | 985.9 | 708.4 | 1049.8 | 476.5 | 499.6 | 485.8 | 253.2 |
| Unknown 33 | 38.85 | 566.3466 | 855.2 | 681.3 | 1685.1 | 185663.0 | 5198.7 | 493.2 | 304.2 | 792.0 | 823.4 | 484.5 | 164.0 | 262.6 |
| Peak 22 | 39.04 | 313.2384 | 19165.9 | 103063.7 | 359.7 | 267.9 | 287.0 | 49.1 | 113.6 | 302.9 | 1294.7 | 578.7 | 2904.0 | 161.8 |
| Unknown 34 | 39.05 | 321.2420 | 192.4 | 813.4 | 201580.8 | 2679.3 | 378.3 | 0.1 | 112.3 | 48.8 | 144.2 | 141.6 | 113.5 | 130.7 |
| Peak 23 | 39.06 | 313.2384 | 124708.9 | 519117.9 | 1101.6 | 182.1 | 779.2 | 565.6 | 328.1 | 1777.8 | 5062.8 | 3149.0 | 13878.0 | 179.0 |
| Peak 24 | 39.34 | 491.3218 | 596.3 | 2878.3 | 703153.4 | 530499.9 | 39664.6 | 899.6 | 659.0 | 2063.5 | 5513.3 | 601.8 | 1560.2 | 579.8 |
| Unknown 35 | 39.34 | 491.3228 | 176.9 | 574.1 | 160244.8 | 156190.5 | 12360.5 | 121.5 | 349.7 | 505.3 | 1508.6 | 467.6 | 504.6 | 307.4 |
| Unknown 36 | 39.36 | 505.3017 | 374.1 | 337.3 | 873.7 | 113515.1 | 718.6 | 124.3 | 95.3 | 105.2 | 502.6 | 486.6 | 240.8 | 86.1 |
| Peak 25 | 39.59 | 483.2735 | 1106.2 | 858.0 | 4305.2 | 900121.9 | 124737.0 | 521.0 | 556.4 | 231.5 | 798.5 | 892.2 | 482.3 | 249.6 |
| Unknown 37 | 39.72 | 653.3228 | 653.1 | 2675.6 | 350.6 | 188139.5 | 31123.6 | 620.6 | 613.5 | 3264.3 | 521.9 | 550.9 | 1425.9 | 671.7 |
| Unknown 38 | 39.87 | 531.3177 | 239.0 | 311.1 | 301.5 | 104100.0 | 2573.7 | 128.6 | 80.5 | 121.8 | 115.8 | 680.0 | 224.2 | 52.8 |
| Unknown 39 | 39.99 | 665.3224 | 292.2 | 184.4 | 159.2 | 117110.5 | 688.1 | 191.6 | 124.1 | 177.1 | 207.2 | 226.3 | 147.8 | 77.5 |
| Peak 26 | 40.13 | 400.3064 | 897.8 | 14197.9 | 311330.8 | 8051.5 | 718.3 | 117.7 | 157.1 | 32.0 | 9910.0 | 7846.4 | 15199.1 | 11925.9 |
| Unknown 40 | 40.15 | 400.3071 | 76.1 | 996.1 | 208671.6 | 7353.5 | 243.1 | 0.1 | 96.8 | 63.7 | 254.6 | 95.0 | 0.1 | 33.9 |
| Peak 27 | 40.20 | 474.9901 | 1710.9 | 21416.7 | 408.8 | 112.0 | 258.6 | 675.6 | 837.8 | 997.2 | 51664.3 | 5564.5 | 717.0 | 363.4 |
| Unknown 41 | 40.44 | 342.3019 | 56.3 | 711.9 | 74592.9 | 20703.4 | 283.8 | 57.5 | 22.5 | 46.3 | 400.4 | 588.3 | 12.4 | 29.7 |
| Unknown 42 | 40.48 | 635.3655 | 302.8 | 890.0 | 61961.9 | 8942.7 | 343.5 | 205.4 | 196.1 | 274.5 | 220.3 | 1012.3 | 249.1 | 106.3 |

| | | | | | | | | | | | | | | |
|---|---|---|---|---|---|---|---|---|---|---|---|---|---|---|
| Unknown 43 | 41.21 | 301.2177 | 666.8 | 429.5 | 70505.0 | 59119.1 | 395.1 | 152.4 | 169.3 | 78.1 | 73.7 | 77.4 | 0.1 | 91.3 |
| Unknown 44 | 41.45 | 621.4368 | 157.5 | 98.1 | 55852.7 | 15226.1 | 3801.9 | 209.4 | 88.3 | 113.7 | 69.9 | 79.1 | 111.9 | 114.5 |
| Unknown 45 | 41.53 | 310.2753 | 88.6 | 4358.9 | 60284.0 | 0.1 | 0.1 | 64.6 | 0.1 | 0.1 | 41.6 | 19111.3 | 24.7 | 28.5 |
| Unknown 46 | 41.54 | 583.3165 | 73.3 | 141.2 | 133.9 | 71040.6 | 29961.5 | 79.2 | 42.2 | 94.1 | 126.8 | 91.0 | 163.9 | 15.8 |
| Peak 28 | 41.66 | 297.2442 | 1357.1 | 2113.6 | 35374.6 | 328816.4 | 5061.8 | 275.6 | 315.2 | 583.0 | 845.9 | 2517.1 | 738.4 | 657.5 |
| Unknown 47 | 41.92 | 431.3213 | 66.3 | 3518.8 | 56182.1 | 314.2 | 46.6 | 29.2 | 28.1 | 46.9 | 118.1 | 8059.5 | 4469.9 | 7583.6 |
| Unknown 48 | 42.12 | 607.2562 | 211.5 | 47.3 | 47803.5 | 7617.0 | 294.5 | 42.5 | 54.3 | 44.9 | 23.1 | 93.5 | 22.7 | 42.3 |
| Unknown 49 | 42.25 | 431.3212 | 145.0 | 758.0 | 55734.8 | 163.1 | 37.5 | 17.3 | 11.0 | 12.1 | 57.9 | 5008.1 | 3886.4 | 2803.0 |
| Unknown 50 | 42.35 | 303.2332 | 219.7 | 2343.2 | 50292.9 | 30303.9 | 337.7 | 9.2 | 40.6 | 17.5 | 26.0 | 82.3 | 55.2 | 91.2 |
| Unknown 51 | 42.44 | 341.2690 | 51799.5 | 4703.2 | 244.8 | 173.8 | 76.8 | 112.9 | 40.5 | 132.4 | 0.1 | 694.7 | 163.9 | 125.9 |
| Unknown 52 | 42.69 | 607.2563 | 80.7 | 163.7 | 53133.4 | 34519.4 | 2269.6 | 66.0 | 6.5 | 18.7 | 0.1 | 22.9 | 15.7 | 17.2 |
| Unknown 53 | 43.11 | 313.2735 | 15935.5 | 10502.9 | 7970.8 | 26899.8 | 123.3 | 155.0 | 28.4 | 37.3 | 184.0 | 45608.1 | 213641.5 | 18189.1 |
| Unknown 54 | 43.19 | 637.2669 | 146.5 | 59.2 | 21888.2 | 56984.5 | 178.6 | 42.4 | 12.7 | 22.7 | 21.1 | 0.1 | 49.6 | 0.1 |
| Unknown 55 | 43.38 | 473.2837 | 576.2 | 1286.3 | 694.7 | 628.1 | 397.3 | 18570.0 | 28576.0 | 21437.5 | 28609.5 | 89064.5 | 51727.8 | 19743.6 |
| Unknown 56 | 43.42 | 607.2565 | 76.1 | 0.1 | 573.3 | 54405.5 | 102.6 | 13.0 | 8.9 | 26.3 | 20.7 | 0.1 | 48.9 | 34.2 |
| Peak 29 | 43.53 | 327.2906 | 45265.4 | 140619.6 | 51488.2 | 107516.0 | 149.9 | 107.2 | 102.0 | 165.5 | 180.9 | 3562.8 | 154544.1 | 5393.6 |
| Peak 30 | 43.86 | 433.3328 | 8015.9 | 224747.7 | 2545.2 | 439.1 | 880.9 | 2621.4 | 581.2 | 1059.2 | 1377.4 | 600670.5 | 664116.4 | 243483.8 |
| Unknown 57 | 43.87 | 785.5042 | 0.1 | 91.4 | 53670.8 | 3799.8 | 93943.5 | 194.2 | 45.5 | 60.5 | 7.5 | 145.2 | 0.1 | 0.1 |

| | | | | | | | | | | | | | | |
|---|---|---|---|---|---|---|---|---|---|---|---|---|---|---|
| Unknown 58 | 43.90 | 687.4328 | 577.7 | 545.2 | 964.8 | 82891.5 | 1020.8 | 368.9 | 69.5 | 0.1 | 105.2 | 556.2 | 7289.3 | 570.8 |
| Unknown 59 | 44.36 | 719.4591 | 608.4 | 447.5 | 83713.8 | 20011.7 | 344.4 | 193.2 | 83.8 | 49.2 | 621.6 | 510.7 | 264.2 | 132.1 |
| Activity Score | - | - | 139 | 80 | 26 | 22 | 0.1 | 20 | 25 | 48 | 120 | 12 | 79 | 0.1 |

**S7:** Schistosomicidal Activity in Adult Worms: Animal care and monitoring.

Cercariae inoculation: a combination of 2 anesthetics, quetamine 125 mg/Kg b.w. (10%, 0.1 g/ml) and xylazine 5 mg/Kg b.w. (2%, 0.02 g/ml) is administrated subcutaneously in the back.
Euthanasia: according to the CEUAIBu recommendations: animals are euthanized by administration of quetamine 375 mg/Kg b.w. and xylazine 15 mg/Kg b.w. via i.p.
Expected adverse effects: weight loss of up to 10% - animals with weight losses above 10% are separated for observation.
Non-expected adverse effects: blindness, weight loss above 20%, fighting. Animals showing these effects are separated from the group.
Observation: all the animals are placed in cages with environmental enrichment to avoid eventual stress.
